# Physiological Changes and Pathological Pain Associated with Sedentary Lifestyle-Induced Body Systems Fat Accumulation and Their Modulation by Physical Exercise

**DOI:** 10.3390/ijerph182413333

**Published:** 2021-12-17

**Authors:** Enrique Verdú, Judit Homs, Pere Boadas-Vaello

**Affiliations:** 1 Research Group of Clinical Anatomy, Embryology and Neuroscience (NEOMA), Department of Medical Sciences, University of Girona, 17003 Girona, Spain; jhoms@euses.cat; 2 Department of Physical Therapy, EUSES-University of Girona, 17190 Salt, Spain

**Keywords:** obesity, overweight, pathological pain, adipose tissue and body systems crosstalking, physical exercise, cytokines, chemokines

## Abstract

A sedentary lifestyle is associated with overweight/obesity, which involves excessive fat body accumulation, triggering structural and functional changes in tissues, organs, and body systems. Research shows that this fat accumulation is responsible for several comorbidities, including cardiovascular, gastrointestinal, and metabolic dysfunctions, as well as pathological pain behaviors. These health concerns are related to the crosstalk between adipose tissue and body systems, leading to pathophysiological changes to the latter. To deal with these health issues, it has been suggested that physical exercise may reverse part of these obesity-related pathologies by modulating the cross talk between the adipose tissue and body systems. In this context, this review was carried out to provide knowledge about (i) the structural and functional changes in tissues, organs, and body systems from accumulation of fat in obesity, emphasizing the crosstalk between fat and body tissues; (ii) the crosstalk between fat and body tissues triggering pain; and (iii) the effects of physical exercise on body tissues and organs in obese and non-obese subjects, and their impact on pathological pain. This information may help one to better understand this crosstalk and the factors involved, and it could be useful in designing more specific training interventions (according to the nature of the comorbidity).

## 1. Introduction

A sedentary lifestyle may trigger obesity, since the lack of physical activity or inactivity could lead to weight gain, and a sedentary lifestyle is highly associated with abdominal and visceral fat excess [1,2,3,4,5,6]. In the United States (U.S.), about 75% of adults older than 20 years meet the criteria for being overweight or obese; 42% are obese, and 9% have morbid or severe obesity [7]. As for the European Union (EU), about 52.7% of the population aged 18 and over are overweight and about 20% are obese [8]. In the U.S., it is estimated that the medical costs of obesity exceed USD 275 billion annually [9]. Considering that the U.S. total health care expenditure reached USD 3.3 trillion in 2016 [10], the obesity-related medical costs represented 8.3% of the total health budget. In Europe, the situation is not much better. It is estimated that, in the EU, the health expenditure associated with overweight and obesity in the period 2020–2050 will be approximately 5% [11]. Hence, these available data indicate that between 20 and 40% of industrialized western populations would be obese, and the related medical costs may represent between 5 and 8% of health budgets.

The World Health Organization (WHO, Geneva, Switzerland) defines obesity as an “abnormal or excessive fat accumulation that presents a risk to health”, commonly classified by the body mass index (BMI). The fundamental cause of obesity is an energy imbalance between calories consumed and calories expended, due to consumption of foods rich in energy, which mainly contain sugars and fats, as well as an increase in physical inactivity due to the increasingly sedentary nature of many forms of work, changing modes of transportation, and increasing urbanization [12]. The excess fat accumulated in different tissues, organs, or body systems, leads to structural and functional human body changes. Several studies suggest that the fat distribution pattern in the human body is associated with the risk of suffering from various obesity-related pathologies. In men, obesity associates with fat accumulation in the abdomen and in the upper part of the body, whereas in women, it affects the gluteofemoral region and the lower part of the body, although as the latter gain weight, they become more likely to develop both upper-body and abdominal fat. [13]. The accumulation of abdominal fat is associated with the development of various disorders, such as atherogenic profile, arterial stiffness, elevated fibrinogen levels, hypertension, insulin resistance, hyperinsulinemia, glucose intolerance, arthritis, irregular menstruation, and gallbladder disorders [14,15,16,17,18,19,20,21,22,23,24,25,26,27,28]. Research shows that physical exercise can reverse part of these obesity-related pathologies by decreasing the availability of free fatty acids and improving the insulin sensitivity of glucose metabolism [29], reducing hypertension [30], decreasing arterial stiffness [31], reducing both fibrinogen and fibrinolytic biomarkers [32], relieving arthritis [33,34], and improving menstrual issues [35]. In addition, exercise could lead to lower body fat and weight [36,37,38,39,40,41,42,43,44,45], which, consequently, could help one deal with the above-mentioned obesity comorbidities.

In addition to obesity-induced disorders, clinical evidence suggests that pain conditions development in obese subjects, such as low back pain [46,47,48,49,50,51,52,53,54,55,56], musculoskeletal pain [57,58,59,60,61,62,63], osteoarthritis pain [64,65,66], knee pain [67,68,69,70], hip pain [71], foot pain [72], spine-related pain [73], migraine and/or headache [74,75,76,77,78,79,80], orofacial pain [81,82], and postsurgical pain [83]. Research shows that physical exercise may relieve obesity-induced pain in affected subjects [84,85,86,87,88,89].

Overall, obesity is a health concern characterized by excessive body fat accumulation and by a set of comorbidities, which may be associated with structural and functional changes in tissues, organs, and body systems. Physical exercise, which reduces body fat, alleviates some of these obesity-related comorbidities, including pathological pain. In this context, the objectives of this work are to review: (i) the structural and functional changes suffered by tissues, organs, and body systems, due to the accumulation of fat in obesity, emphasizing the crosstalk between fat and body tissues; (ii) the crosstalk between fat and body tissues triggering pain exacerbation; and (iii) the effects of physical exercise on body tissues and organs in obese and non-obese subjects, and their impact on pathological pain relief.

## 2. Obesity-Induced Changes in Tissues, Organs, and Body Systems: Crosstalk between Fat and Body Tissues

Before describing the effects of physical exercise on tissue crosstalk (in a “fat excess” context), in this section, we review preclinical and clinical evidence of the accumulation of fat in different tissues, organs, and body systems in obese subjects, the humoral factors synthesized and released by these adipose tissues, and how these factors influence the physiology of the tissues, organs, and systems where they accumulate. At the end of this section, we review the deposit of body adipose tissue, and how this deposit of fat confers to an increased risk of metabolic diseases in obese subjects, such as metabolic syndrome, metabolically healthy obesity, and metabolically unhealthy obesity, indicating, for each of them, the characteristics of their adipose tissue.

### 2.1. Skeletal Muscle

Obesity causes an accumulation of fat in skeletal muscles, mainly inside muscle cells occupying a central region in the muscle fiber. This accumulation of lipid drops in obese subjects affects the three types of muscle fibers (fibers I, IIA, and IIB) [90,91]. Obesity causes dysregulation of the metabolism of these accumulated lipids in skeletal muscle cells, which leads to a significant decrease in the degree of lipid unsaturation [92]. This lipid unsaturation reduction in muscle cells of obese subjects may result from increased lipid peroxidation. Moreover, in the Otsuka Long-Evans Tokushima fatty (OLETF) rat, an animal model of obesity, the levels of lipid peroxidation in skeletal muscle fibers were significantly higher in sedentary—rather than runner—animals, suggesting that muscle lipid peroxidation increases in sedentary obese subjects [93]. In turn, the accumulation of lipid droplets within skeletal muscle fibers and the consequent increase in lipid peroxidation in these cells may be involved in the development of skeletal muscle insulin resistance in obese sedentary subjects [94]. Under physiological conditions, insulin secreted by an increase of glucose in the blood, causes upregulation of glucose transporter type 4 (GLUT4) expression in muscle cells via activation insulin–tyrosine–kinase receptors, phosphorylation of insulin receptor substrates 1 and 2 (IRS1, and IRS2), activation of phosphatidylinositol 3-kinase (PI3K), and atypical protein kinase C (PKCζ) [95]. Overexpression of GLUT4 transporters in the membrane of skeletal muscle fibers promotes glucose reuptake in skeletal muscle fiber, and immediately, glucose is phosphorylated by hexokinase to prevent cell leakage. Phosphorylated glucose can be stored in the form of muscle glycogen or used in oxidative glycolysis for energy production [96]. In contrast, when elevated insulin levels produce an attenuated biological response, this is defined as insulin resistance [97]; this would occur in obese individuals. Under obesity conditions, the accumulated lipids in skeletal muscle cells promotes muscle cells to obtain energy through lipid oxidation, resulting in the production of 4-hydroxy-2-hexenal (4-HHE), a lipid aldehyde that affects insulin-induced IRS1/IRS2 phosphorylation [98]. Moreover, another lipid aldehyde that is produced from lipid peroxidation in skeletal muscle cells is 4-hydroxy-2-nonenal (4-HNE), which is known to causes a significant reduction of IRS1 phosphorylation in skeletal muscle cells, and increases the ROS production promoting lipid peroxidation in these cells [99]. Altogether, under obesity conditions, the accumulation of lipids in skeletal muscle cells favors the use of this energy source for obtaining ATP molecules through the metabolism of lipid oxidation. Two products of this lipid oxidation (4-HHE and 4-HNE) interfere in the phosphorylation of IRS1 and, thus, in the final translocation of GLUT4 transporters in the plasma membrane of the skeletal muscle cell. Hence, in an obese person, after food intake, the insulin plasma increase will not result in proper GLUT4 overexpression in skeletal muscle cells because of the interference of these lipid aldehydes in the signaling pathway responsible for GLUT4 expression, leading to the insulin resistance of the skeletal muscle observed in obese subjects.

In the same context, other physiological changes may explain insulin resistance in obese subjects, such as the increase of fatty acid synthase (FAS) and choline/ethanolamine phosphotransferase 1 (CEPT1) enzymes activity, and/or expression. In mice, it was reported that high-fat diet feeding increases FAS protein expression and enzyme activity in skeletal muscle fibers of obese mice that also show insulin resistance. By using FAS-KO animals that present FAS deficiency in the skeletal muscles, but not in the liver, heart, or pancreas, it was observed that FAS deletion causes AMPK (AMP-activated protein kinase) activation in skeletal muscle cells of mice treated with a high-fat diet. As AMPK promotes skeletal muscle insulin sensitivity, these results suggest that FAS expression inhibits AMPK and, thereby, generates insulin resistance in skeletal muscle fiber [100]. In addition, it is well known that FAS is located in plasmatic membrane and in sarcoplasmic reticulum (SR), promoting SERCA activity, allowing the entry of calcium ions into the SR and, thereby, facilitating the process of muscle relaxation. In FAS-KO mice, it was reported that FAS deficiency decreased SERCA activity by altering the phospholipid composition of the SR, which resulted in increased cytosolic calcium and AMPK activation. FAS-KO mice showed lower insulin resistance than wild type mice, but their skeletal muscles showed higher weakness than wild type animals [100]. These findings suggest that FAS activity is associated with insulin resistance in skeletal muscles. In the same line, a high-fat diet increases choline/ethanolamine phosphotransferase 1 (CEPT1) expression in mouse skeletal muscle, and increases phosphatidylcholine (PC) and phosphatidylethanolamine (PE) abundance in skeletal muscle SR, maintaining the SERCA activity. Similarly, in absence of CEPT1 (CEPT1-KO mice), altered PC:PE content decreased SERCA activity, increased cytosolic calcium, causing muscle weakness, but increased muscle insulin sensitivity, via calcium activation of AMPK [101]. These findings suggest that CEPT1 is also related to insulin resistance.

One of the direct consequences of insulin resistance in muscle fiber is that muscle mitochondria must use free fatty acids (FFA) in plasma as an energy source, to maintain, among other muscle functions, muscle contraction, and muscle strength during exercise and daily activity of the obese subject. However, ATP production by beta-oxidation of FFA is lower than oxidation of glucose, about 14 and 36 ATP molecules, respectively. Therefore, the insulin resistance caused by obesity forces skeletal muscles to use the beta-oxidation of free fatty acids and other lipids with a lower yield of ATP molecule production, and with it, less production of muscle force and muscle fatigue occurs more quickly due to the consumption of muscle glycogen stores [102]. Using a zebrafish model, it was shown that obesity causes a decrease in sustained swimming speed, maximum locomotor capacity, and isolated muscle isometric stress that are not reversed by weight loss, whereas obesity increases resting metabolic rates and decreases maximal metabolic rate that are reversed by weight loss [103].

Finally, intermuscular adipose tissue (IMAT) marbled within skeletal muscles is also related to skeletal muscle insulin resistance. IMAT increases in an obese subject, it is preferent in the lower-extremity muscles [104,105]. Using fat (intermuscular, subcutaneous, and visceral adipose tissues) and muscle biopsies of human subjects, it was reported that factors secreted from IMAT decrease insulin sensitivity in vitro with a potency similar to visceral adipose tissue (VAT), because both adipose tissues induce 1,2-diacylglycerol (DAG) accumulation in the skeletal muscles. The insulin-IMAT resistance is related to a decrease of mitochondrial function and lipid oxidation, which contribute to enhanced release of free fatty acids into interstitial fluid surrounding muscle [106]. In both mice and humans, DAG accumulation in skeletal muscle cells promote the PKC-theta activation and the subsequent decrease in insulin signaling at the level of the insulin receptor substrate (IRS)-1 tyrosine phosphorylation [107,108,109,110,111]. IMAT secretes cytokines and chemokines that contribute to insulin resistance of skeletal muscles, including monocyte chemoattractant protein-1 (MCP-1) [106,112], and contributes to macrophages and lymphocyte infiltration in skeletal muscles of obese subjects [113]. In pigs, it was reported that IMAT secretes several micro-RNAs related to insulin resistance and inflammation of skeletal muscles [114].

### 2.2. Cardiovascular System

Perivascular adipose tissue (PVAT) is located outside the adventitial layer and surrounds most of the systemic blood vessels, with the exception of the blood vessels of the central nervous system [115,116,117,118]. In small vessels and microvessels, PVAT is integrated in the vascular wall without laminar structures that separate PVAT from the adventitia layer [117,118]. Structurally, PVAT consists of adipocytes, fibroblasts, stem cells, eosinophils, T lymphocytes, and macrophages [119,120]. In large vessels, between PVAT and the vascular wall, a network of elastic and collagen fibers, fibroblasts, vasa vasorum, and sympathetic nerve fibers is observed [121]. PVAT releases several factors that reach medial and endothelial layers of blood vessels by direct diffusion, through the vasa vasorum, and through the network of collagen ducts that connect the media layer with the adventitia. These factors regulate the physiology/pathophysiology of blood vessels, including the vascular tone, inflammation of vascular wall, vascular remodeling, and atherosclerosis [118,119,120,122,123,124].

The factors released by PVAT can be classified into: (i) vasodilator factors, such as hydrogen peroxide (H2O2), hydrogen sulfide (H2S), methyl palmitate, nitric oxide, adiponectin, leptin, apelin, omentin, adrenomedullin [118,120,124,125]; (ii) vasoconstrictor factors including angiotensin-II, chemerin, adiponectin, and apelin [118,120,122,124]; (iii) inducing factors of vascular remodeling, such as micro-RNAs (e.g., miR-221–3p), growth factors (e.g., thrombospondin-1, serpin-E1, TGF-β, PDGF-BB, VEGF, bFGF, hepatocyte growth factor, insulin-like growth factor-binding protein-3), visfatin, and leptin that promote neointima formation, and proliferation of vascular smooth muscle cells after vascular injury and/or atherosclerosis [122,126,127,128]; (iv) inhibitory factors of vascular remodeling including adiponectin, and C1q/TNF-related protein 9 (CTRP-9) [124,129,130]; (v) anti-inflammatory factors secreted under physiological conditions such as adiponectin, IL10, prostacyclin [123]; (vi) pro-inflammatory factors released under vascular injuries (e.g., atherosclerosis, hypertension) including leptin, resistin, visfatin, IL6, TNF-alpha, RANTES, MCP-1/CCL2, CCL3, CCL5, reactive oxygen species (ROS) [123]; (vii) pro-atherogenic factors, such as IL17 [119,131]; (viii) anti-atherogenic agents, including nitric oxide, H2S, adiponectin, vaspin, apelin, omentin, chemerin, leptin, resistin, lipocalin-2 (LCN2), and visfatin [119,120,132,133].

Animals consuming high-fat diets (HFDs) rapidly gain weight and become obese subjects. Obesity causes an increase of PVAT and a hypertrophy of adipocytes around the blood vessels, and an increase of eosinophils, macrophages, T-cells, MCP-1, and TNF-alpha expression, but endothelial nitric oxide synthase expression decreases in these obese animals [132,134,135,136]. In humans, PVAT samples from obese subjects showed that the expression of MCP1/CCL2, CCL8, IL1beta, and IL6 proteins were upregulated, suggesting that obesity triggers upregulation of pro-inflammatory factors secreted by PVAT [137]. In turn, it is well known that CCL2, TNF-alpha, IL1beta, and IL6 are pro-inflammatory factors are involved in atherosclerotic plaque formation; that is, they favor vascular walls lipids deposition [138]. In addition, MCP1/CCL2 secreted by PVAT it is a factor that favors the attraction and recruitment of monocytes/macrophages towards the vascular wall, and monocyte–endothelial cell adhesion plays a key role in the initiation of atherosclerosis. After adhering to the endothelial cells, monocytes penetrate through endothelial cells into the subendothelial space, transforming in macrophages, and participating in scavenging lipoprotein particles and turn into foam cells, which present cytoplasmic and membrane-bound droplets, resulting in more accumulation of lipoproteins in the subendothelial space [139,140]. Several studies suggest that TNF-alpha contributes to eosinophil recruitment [141,142,143], and eosinophils attracted to PVAT-secreted TNF-alpha contribute to atherosclerotic lesion formation by activating the endothelial cell [144]. In addition, eosinophils interact directly with platelets and induce eosinophil activation and the release of eosinophil extracellular traps that stabilizes the thrombus [144]. In summary, obesity causes an increase in perivascular adipose tissue (PVAT) that releases pro-inflammatory factors that interact with the blood vessel walls cells, favoring the recruitment of monocyte–macrophages and eosinophils and the generation of atherosclerotic plaques. That is, the crosstalk between the perivascular adipose tissue and the wall of the blood vessels results in atherosclerotic lesions in the blood vessels of the obese subjects.

On the other hand, epicardial adipose tissue (EAT) is a visceral thoracic fat depot located along the large coronary arteries, on the surface of the ventricles and the apex of the heart. This EAT constitutes about 20% of the total ventricular weight in the human heart. Compared to other fat depots, the number of adipocytes per gram of EAT is higher, but their size is smaller. In comparison with subcutaneous adipose tissue, EAT shows higher levels of saturated fatty acids and lower levels of unsaturated fatty acids, and higher levels of inflammatory mediators, including IL1beta, IL6, TNF-alpha, MCP1/CCL2, CCL3, CCL4, CCL5, CCL11, CCL18, and CCL21. Moreover, EAT express higher levels of resistin, but lower levels of adiponectin and leptin [145,146,147]. It is well known that EAT thickness increases in obese subjects [148,149,150,151,152,153], and in these subjects, the levels of factors secreted by EAT, such as TNF-alpha, IL6, leptin, and free fatty acids, increase in comparison with non-obese subjects [154,155]. As for free fatty acids secreted by EAT in obese subjects, the metabolic signature of EAT is dominated by an increase of phosphatidylglycerol, phosphatidylcholine, and phosphatidylethanolamine [156]. In this context, it is known that TNF-alpha and IL-1beta reduce contractile function and impair cardiac chemomechanical energy transduction in human myocardium [157]. Moreover, activin-A, TNF-alpha, IL6, and MCP1/CCL2 secreted by EAT contributes to myocardial fibrosis [158,159,160]. In addition, elevated free fatty acids levels may impair cardiac function and cardiac dysfunction might result from myocardial insulin resistance with significant changes to PI3K-Akt-GLUT4 and AMPK-eNOS signaling pathways, with increasing free fatty acids levels [161]. Altogether these findings suggest that factors secreted by EAT causes changes in cardiomyocytes, affecting their physiology.

Pericardial adipose tissue (PAT) covers approximately 80% of the heart and constitutes 20–50% of the heart weight in humans. PAT is located outside the pericardium on the external surface of the parietal pericardium [145]. It was shown that, in obese subjects, the PAT increases [162,163], and in obese Lee-Sung mini pigs, it secretes higher levels of TNF-alpha, TGF-beta, platelet-derived growth factor (PDGF), leptin, and resistin [164]. Moreover, in these mini pigs, the PAT contributes to cardiac fibrosis development [165]. In humans, PAT also contributes to cardiac insulin resistance and left ventricular systolic dysfunction in obese individuals [166,167]. TGF-beta promotes cardiac fibrosis, the formation of cardiac fibroblasts into myofibroblasts. Activated fibroblasts differentiate into myofibroblasts, which increases their ability to produce ECM proteins. This change leads to increased myocardial stiffness and, ultimately, cardiac dysfunction and heart failure [168]. Similarly, PDGF also induces cardiac fibrosis [169], and as mentioned above, TNF-alpha contributes to reduce contractile function of myocardium [157]. Resistin and leptin promote cardiac hypertrophy [170,171,172], whereas leptin is also implicated in the regulation of cardiac contractility via a local nitric oxide dependent mechanism [173]. Hence, these findings suggest that several factors secreted by pericardial adipose tissue also influence the physiology of the heart.

### 2.3. Accumulation of Fat in Liver and Pancreas

Excessive fat accumulation within the liver is called hepatic steatosis. There is a positive correlation between obesity and hepatic steatosis, and obese people are four times more likely to develop hepatic steatosis [174,175,176]. Hepatic steatosis is one of the characteristics of non-alcoholic fatty liver disease (NAFLD), with >5% of hepatocytes infiltrated with fat [177]; it is well known that a sedentary lifestyle and a high-fat diet (HFD) promotes NAFLD [178,179]. Consequently, NAFLD is strongly linked to obesity, with a reported prevalence as high as 80% in obese patients and only 16% in individuals with a normal body mass index and without metabolic risk factors [175,180,181]. A change in the nomenclature of NAFLD to metabolic (dysfunction)-associated fatty liver disease (MAFLD) was proposed by expert panels, because multiple metabolic factors contributing to the development and progression of NAFLD and, consequently, MAFLD, is used for hepatic disease related to the presence of metabolic causes. According to a new consensus, NAFL, and nonalcoholic steatohepatitis (NASH) is abolished and both phenotypes are placed under the umbrella of MAFLD [182,183,184]. Thus, MAFLD is defined as liver fat accumulation (hepatic steatosis), combined with the presence of overweight/obesity or T2DM, or at least two metabolic risk factors, including (i) waist circumference ≥102 cm in men or ≥88 cm in women (≥90/80 cm in Asian); (ii) triglyceride (TG) levels ≥150 mg/dL or treatment for TGs; (iii) high-density lipoprotein cholesterol levels <40 mg/dL in men and <50 mg/dL in women or treatment for dyslipidemia; (iv) systolic blood pressure ≥130 mm Hg or diastolic pressure ≥85 mm Hg or treatment for arterial hypertension; (v) prediabetes; (vi) homeostasis model assessment of insulin resistance (HOMA-IR) ≥2.5; (vii) high-sensitivity C-reactive protein levels >2 mg/L [183].

A diet consisting of excessive caloric intake composed of fats and carbohydrates, a sedentary lifestyle, and visceral adipose tissue increases are major contributors to the pathogenesis of MAFLD. Insulin resistance also contributes to MAFLD, which promotes lipolysis and de novo lipogenesis. Moreover, dysbiosis of gut microbiota, by increasing gut permeability, leads to increased absorption of fatty acids and bacterial translocation, thus promoting inflammation. It is worth mentioning that genetic predisposition also contributes to the pathogenesis of MAFLD. These factors first result in fat accumulation into the hepatocytes and subsequently trigger oxidative stress, endoplasmic reticulum stress, and inflammation pathways, leading to both hepatocytes apoptosis and fibrosis [185]. Hepatic fat accumulation results from an imbalance between lipid acquisition and lipid disposal, which are regulated through four major pathways: uptake of circulating lipids, de novo lipogenesis (DNL), fatty acid oxidation (FAO), and export of lipids in very low-density lipoproteins (VLDL) [186,187]. The uptake of circulating fatty acids by the liver dependent on fatty acid transporters such as fatty acid transport proteins (e.g., FATP2 and FATP5), a cluster of differentiation 36 (CD36), and caveolins located in the hepatocyte plasma membrane. Following uptake, hydrophobic fatty acids do not diffuse freely in the cytosol and must instead be shuttled between different organelles by specific fatty acid binding proteins (e.g., FABP1). FABP1 facilitates the transportation, storage, and utilization of fatty acids and their acyl-CoA derivatives, and may exert a protective effect against lipotoxicity by binding otherwise cytotoxic free fatty acids, facilitating their oxidation or incorporation into triglycerides [188]. Consequently, FAB1 play an important role in the accumulation of circulating lipids in triglycerides inside hepatic cells, causing hepatic fat accumulation and MAFLD. In addition, hepatic cells synthesize new fatty acids from acetyl-CoA that is converted to malonyl-CoA by acetyl-CoA carboxylase (ACC); malonyl-CoA is then converted to palmitate by fatty acid synthase (FASN). New fatty acid may then undergo a range of desaturation, elongation, and esterification steps before ultimately being stored as triglycerides or exported as VLDL particles. Consequently, increased lipogenesis causes hepatic steatosis and/or hypertriglyceridemia [187,188]. It is well known that hepatic new lipogenesis is regulated by two transcription factors: sterol regulatory element-binding protein 1c (SREBP1c) and carbohydrate regulatory element-binding protein (ChREBP) [189,190,191]; the expressions of both transcription factors are upregulated in patients with MAFLD [192,193,194]. These findings suggest that liver de novo lipogenesis is promoted in MAFLD, causing triglyceride storage in liver cells and the development of hepatic steatosis.

The increase in intrahepatic triglycerides (IHTG) in obese subjects with MAFLD has various functional effects in the liver, such as a reduction in hepatic insulin extraction, and increased triglycerides, alanine aminotransferase (ALT), and aspartate aminotransferase (AST) in plasma [195,196,197]. In addition, it was reported that serum irisin levels reduced gradually with the increase of IHTG contents in obese adults with MAFLD [198]. The level of serum fibroblast growth factor-21 (FGF-21), a hepatokine secreted by liver, increased gradually with the increase of IHTG contents in obese subjects with MAFLD [199]. Irisin inhibits hepatic cholesterol synthesis [200] and their decrease in obese adults with MAFLD suggest that hepatic cholesterol synthesis is potentiated in these patients. On the other hand, hepatocyte cultures treated with FGF-21 showed a reduction of mRNA and protein SREBP1c expression [201], suggesting that lipogenesis in hepatic cells is reduced by FGF-21. However, FGF-21 facilitates fatty acid binding protein (FABP) expression [202]. These findings suggest that diffuse factors secreted by fat liver have paracrine/autocrine effects by enhancing the entry of circulating fatty acids to hepatocytes and promoting the hepatic cholesterol synthesis, thus increasing the accumulation of lipids in the fat liver. In other words, liver fat itself enhances diffusible factors that enhance the accumulation of lipids in hepatocytes.

In addition to the accumulation of fat in the liver, obese subjects may accumulate it in the pancreas [203,204,205,206]. For instance, the prevalence of a fatty pancreas is about 16–35% in Asian populations [207,208], highly associated with nonalcoholic fatty pancreas disease (NAFPD), which is specifically defined as pancreatic fat accumulation in association with obesity in the absence of significant alcohol consumption [209]. In contrast to the liver, fatty infiltration of the pancreas spares the acini (exocrine function) and islets of Langerhans (endocrine function), and preferentially accumulates within the pancreatic interstitial septa [210]. The death of acinar cells and their substitution by adipocytes (“fatty replacement”) and fatty accumulation (“fatty infiltration”) are the two main mechanisms leading to fatty accumulation in the pancreas [209]. “Fatty replacement” is characterized by the death of pancreatic acinar cells and their respective replacement by adipocytes, which can be caused by several conditions. The main factors for pancreatic cell death include genetic factors, such as fibrosis, or clinical features, such as excessive alcohol consumption, viral infections, iron overload (mainly represented by hemochromatosis), use of medication (corticosteroids), or obstruction of the pancreatic duct (chronic obstructive pancreatitis). ‘‘Fatty infiltration’’ occurs when there is an infiltration of adipocytes in the pancreatic tissue. The infiltration of pancreatic fat initially causes hypertrophy and hyperplasia of the pancreatic cells, resulting in clinical conditions, such as insulin resistance, beta-cell dysfunction, and diabetes mellitus type 2 [211].

Peri-pancreatic adipose tissue releases several factors that modulate beta-cell physiology including CXCL-1, CXCL-2, CXCL-3, CXCL-5/LIX, MIP-1α/CCL3, and VEGF [212]. VEGF secreted by peri-pancreatic adipose tissue plays an important role promoting and maintaining vascularization of pancreatic islets, indispensable to collect insulin secreted by beta-pancreatic cells [213,214]. VEGF also promotes beta-cell proliferation and prevents the development of hyperglycemia [215]. However, increased expression and secretion of VEGF facilitates hypervascularization and infiltration of macrophages in pancreas, which secrete proinflammatory cytokines (e.g., TNF-alpha, IL6) that impair insulin secretion decrease beta-cell mass, and promote hyperglycemia [216]. Increased levels of vascular growth factors (e.g., VEGF, VEGF-C, and VEGF-D) are present in overweight and obese subjects [217]. These findings suggest that obesity causes an overexpression of VEGF that promotes macrophage infiltration in the pancreas and secretion of pro-inflammatory cytokines that affects insulin secretion by beta cells, causing hyperglycemia. It is well known that TNF-alpha inhibits the expression of ATP-binding cassette transporter A1 (ABCA1), a protein related to the insulin secretion by pancreatic beta cells. Thus, TNF-alpha secreted by infiltrated macrophages in the pancreas affect insulin secretion [218]. In contrast, IL6 enhances insulin secretion by increasing glucagon-like peptide-1 (GLP-1) secretion from L cells and alpha cells [219], but the plasma level of IL6 is similar between overweight/obese and non-obese subjects [220]. On the other hand, increased expression and secretion of CXCL-1, -2, -3, and CXCL-5/LIX chemokines by peri-pancreatic adipose tissue do not exert a direct effect on pancreatic β-cell dysfunction [212]. Similar to VEGF, MIP-1α/CCL3 also promotes pancreatic β-cell proliferation [221], as well as the infiltration of macrophages in the pancreas [222], and serum levels of MIP-1α/CCL3 are increased in obese subjects [223]. Dead acinar cells replaced by adipocytes or the infiltration of adipocytes are the two mechanisms of accumulation of pancreatic fat. The peri-pancreatic adipose tissue releases chemokines and VEGF that favor the infiltration of macrophages in the pancreas, immune cells that release cytokines responsible for inhibiting insulin secretion by beta-pancreatic cells and the generation of hyperglycemia in obese subjects.

### 2.4. Visceral, Abdominal, and Subcutaneous Adipose Tissues

Obesity causes an increase of visceral [224,225], abdominal [226,227,228], and subcutaneous adipose tissues [229]. While the visceral adipose tissue (VAT) is the accumulation of adipocytes around viscera, the adipose tissue beneath the skin is the subcutaneous adipose tissue (SAT). The word “viscera” refers to “organs in the cavities of the body” and considering the three main body cavities, VAT involve adipose tissue distributed in these cavities: intrathoracic, intra-abdominal, and intrapelvic cavities. Consequently, VAT is the adipose tissue within the chest, abdomen, and pelvis [230]. On the other hand, subcutaneous adipose tissue (SAT) consists of three types of white adipose tissue (WAT): deposit WAT (dWAT), structural WAT (sWAT), and fibrous WAT (fWAT). The dWAT is located essentially in large depots in the abdominal area (peri-umbilical), formed by non-lobulated adipose tissue defined as metabolic fat by its large lipidic mass and very poor collagenic component, cells are large and few blood vessels are present. The sWAT is located in some areas in the limbs and in the hips including trochanters, sovrapubic area, armpits, inner faces of the knees, thighs, arms, pectoral, and mammary areas. The stroma is fairly well represented, with a good vascularity and adequate staminality. Cells are wrapped by a basket of collagen fibers. The fWAT is a noteworthy fibrous component and can be found in areas where a severe mechanic stress occurs. Adipocytes are therefore smaller, with a thick fibrous shell wrapping them one-by-one [231]. Abdominal adipose tissue is the accumulation of adipocytes in the abdominal area; however, abdominal fat is composed of abdominal subcutaneous fat and intra-abdominal fat [232]. Abdominal subcutaneous fat is in continuity with the dermal tissue and it is usually considered a unique, homogeneous fat deport. In this abdominal region, SAT is composed by two anatomically different layers, located, respectively, above and below a connective plane named fascia superficialis of the fascia of Scarpa, and is usually indicated as superficial and deep adipose tissues [227]. Intra-abdominal fat is composed of visceral or intraperitoneal fat, mainly consisting of omental and mesenteric fat and retroperitoneal fat masses [232,233].

Excess of both VAT and SAT are associated with different abnormalities, including insulin resistance, impaired glucose tolerance, type 2 diabetes, hypertension, heart failure, coronary heart disease, myocardial infarctions, valve diseases, arrhythmias, sleep apnea, chronic obstructive pulmonary disease, stroke, reduced brain size, reduced grey matter, reduced cognitive function, dementia, reduced bone density, polycystic ovary syndrome, and cancer [234,235,236]. In the context of tissue crosstalk, it is worth noting that these adipose tissues secrete several factors that affect the physiology of different tissues, organs, and body systems. It is known that in obese subjects, VAT/SAT adipose tissue expresses mRNA and secretes soluble factors, including cytokines (e.g., TNF-alpha, IL-1beta, IL6, IL7, IL10, IL8, IL12, IL13, IL15, IL18, IL32, IL33, TNF-like weak inducer of apoptosis or TWEAK, RANTES) [237,238,239,240,241,242,243,244,245,246,247,248,249,250,251,252,253,254,255,256], chemokines (e.g., CXCL2, CXCL9, CXCL10, CCL1, MCP1/CCL2, Fractalkine, MIP1, MIP3) [247,249,257,258,259,260,261], osteopontin [262,263], osteoprotegerin [255], interferon-gamma [264], and adipokines (e.g., omentin, adiponectin, visfatin, leptin, adrenomedullin) [249,252,265,266,267,268,269]. Moreover, VAT/SAT adipose tissue also releases micro-RNAs (e.g., miR-181a-5p, miR-23a-3p, miR-199a-3p, miR-193, and miR-126) [270,271,272].

Osteopontin and IL18 are related to insulin resistance in obese subjects [273]. Under chronic heart stresses (e.g., pressure overload, hypertrophy, cardiomyocyte injury), the quiescent cardiac fibro-adipocyte progenitors (FAPs; quiescent stem cells) become activated and are differentiated in two non-cardiac lineages, including intracardiac adipocytes and intracardiac fibroblast. This activation is mediated by osteopontin [274]. Osteopontin also acts on the cardiovascular system, causing arteriosclerosis, angiogenesis, myocardial remodeling [275], valvular stenosis, hypertrophy, myocardial infarction, heart failure [276], aortic disorders, and coronary artery disease [277]. This protein is also related to several renal diseases, including stone formation, tubulointerstitial nephritis, glomerulonephritis, acute ischemic renal injury, interstitial inflammation and fibrosis, hydronephrosis, chronic cyclosporine-induced nephropathy, and lupus nephritis [278]. Moreover, it plays an important role in mineralization and bone resorption [279]. In the gastrointestinal system, osteopontin is associated with several diseases, such as cancer, inflammatory bowel disease, primary sclerosing cholangitis, cirrhosis, nonalcoholic fatty liver diseases, and helicobacter pylori infection [280]. As for the respiratory system, osteopontin is related to fibrosis development, allergic airways diseases, and pulmonary vascular disease [281]. Finally, osteopontin mediates inflammation in ectopic fat depots present at both the cardiac and renal level [279]. It is true that most body organs secrete osteopontin, and osteopontin levels increase in obese subjects [262], but it cannot be ruled out that the osteopontin secreted by visceral and subcutaneous adipose tissue contributes to the generation of these pathologies of different body organs and systems in obese subjects.

As for osteoprotegerin, it is associated with atherosclerotic disease development in obese subjects [282], together with insulin resistance increasing in obese adolescents [283,284]. In contrast, in obese children, an osteoprotegerin level decrease was reported, suggesting that bone formation relative to resorption was reduced in this population, and consequently increased bone fractures [285]. Osteoprotegerin is also associated with cardiovascular pathologies, including vascular calcification [286], atherosclerosis, endothelial dysfunction, and hypertension [287]. This protein increases in subjects with several lung diseases, including asthma [288], lung cancers [289], and pulmonary arterial hypertension [290,291]. Similarly, osteoprotegerin increases after inflammatory bowel disease [292,293] and gastrointestinal carcinoma [292]. Finally, research shows that osteoprotegerin protects against muscular dystrophy [294,295]. These results suggest that several body tissues and organs are targets of osteoprotegerin and, therefore, respond to the release of this protein by different body cells, including subcutaneous and visceral adipose tissue.

As for interferon-gamma (INF-γ) levels, it was shown to be increased in obese subjects [296,297], in obese subjects with asthma [298], and in obese subjects with prediabetes [299]. Directly or indirectly—INF-γ participates in several stages of atherogenesis, including oxidative stress, promote foam cell accumulation, stimulate smooth muscle cell proliferation and migration into the arterial intima, enhance platelet-derived growth factor expression, and destabilize plaque [300]. Evidence suggests that INF-γ levels are increased in subjects with asthma [301,302,303,304]. Non-eosinophilic asthma, where neutrophils are present in the airway lumen, is mediated by type 1 lymphocytes, which secretes interferon-gamma and TNF-alpha. Both factors promote neutrophil IL-6 secretion. This cytokine contributes to the activation of macrophages of adipose tissue in the lungs, which release pro-inflammatory cytokines that contribute to asthma pathogenesis [305]. Altogether, these findings suggest that, directly or indirectly, INF-γ contributes to both specific cardiovascular and lung diseases development.

It is well known that the repertoire of cytokines, chemokines, and adipokines secreted by visceral and/or subcutaneous adipose tissue are involved in asthma pathogenesis, and the reduction of weight by diet, exercise, or bariatric surgery reduces inflammatory activity, and improve asthma and lung function [305]. Obese adipose tissue releases free fatty acids (FFA), adipokines, and many pro-inflammatory chemokines. These factors are known to play a key role in regulating malignant transformation and colon cancer progression [306,307], and inflammatory bowel disease is associated by secretion of adipokines by adipose tissue [308]. The factors secreted by VAT/SAT adipose tissue (e.g., cytokines, chemokines, adipokines, FFA) also contribute to insulin resistance [309,310] and atherosclerosis in obese subjects [311]. Moreover, pro-inflammatory molecules (e.g., IL6, TNF-alpha) secreted by VAT adipose tissue predispose to cardiac dysfunctions (e.g., end-diastolic septum thickness, end-diastolic posterior wall thickness, absolute and indexed left ventricular mass, deceleration time, myocardial performance index, isovolumetric relaxation time) in obese subjects [312]. Finally, it is known that the production of pro-inflammatory cytokines in VAT/SAT adipose tissue is increased in obese patients with chronic kidney disease [313]. These findings suggest that the factors synthesized and secreted by the visceral and subcutaneous adipose tissue generate a state of generalized body inflammation that contributes to the appearance of dysfunctions of the cardiovascular, respiratory, gastrointestinal, and renal systems. Therefore, there is wide crosstalk between the adipose tissue and various organs and body systems.

In parallel, it is known that VAT/SAT adipocytes secrete exosomes containing several micro-RNAs. For instance, the secretion of miR-181a-5p, miR-23a-3p, and miR-199a-3p are related to insulin resistance in obese subjects [271,272]. Moreover, other micro-RNAs, such as miR-193, miR-126, secreted by VAT adipose tissue promote CCL2 secretion by body adipocytes, a chemokine that facilitates monocyte/macrophage infiltration in adipose tissue, cells that in turn release pro-inflammatory cytokines [270]. On the other hand, a reduction of miR-181a-5p release is associated with atherosclerotic plaque formation [314] and downregulation of miR-181a-5p also prevents cerebral ischemic injury [315] and alleviates oxidative stress and inflammation in coronary microembolization-induced myocardial damage [316]. In this context, it is also known that microRNA-23a-3p attenuates oxidative stress injury in a mouse model of focal cerebral ischemia-reperfusion [317] and microRNA-199a-3p protect cardiomyocytes from simulated ischemia-reperfusion injury [318], participating in the regulation of the NOS (NO Synthase)/NO pathway in the endothelium [319]. This micro-RNA inhibits angiogenesis in an in vitro experimental model of diabetic retinopathy [320].

Finally, in obese subjects, the adipocytes from VAT and SAT also express several molecules, such as lipocalin-2 [321,322], tenascin C, toll-like receptor 4 [323], and protein pentraxin 3 (PTX3) [324], which are related to adipose tissue inflammation and secretion of pro-inflammatory cytokines [321,322,323,325,326,327,328,329,330], insulin resistance [325,331], hyperglycemia and glucose intolerance [325,332,333,334], endothelial dysfunction with hypertension [335], cardiovascular dysfunctions [336,337], and atherosclerosis [338,339].

**Figure 1:**
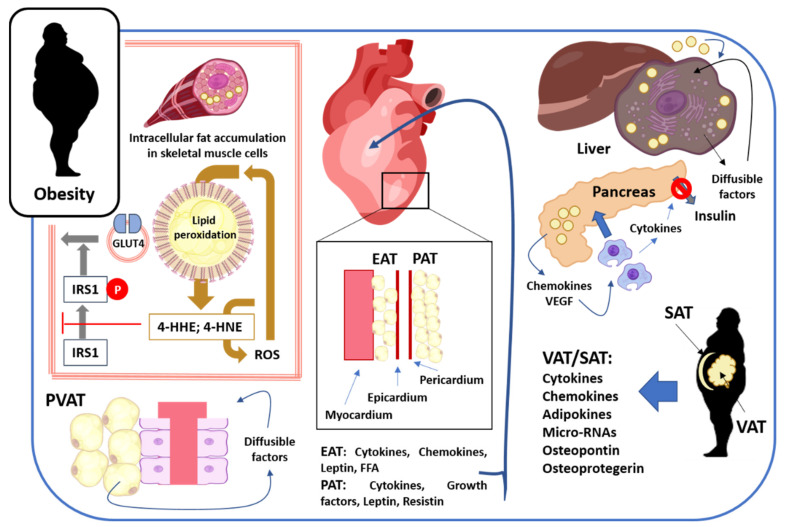
Overview effects of adipose tissue in obese subjects. In skeletal muscle, fat accumulates inside muscle fibers, causing a dysregulation of cellular metabolism and favoring lipid peroxidation. The peroxidation of intracellular lipid droplets triggers the appearance of 4-HNE and 4-HNE, two mediators that interfere with IRS1 phosphorylation, thereby interrupting the transport of vesicles loaded with the GLUT4 transporter to the muscle membrane. Physiologically, insulin, by binding to its receptor, favors IRS1 phosphorylation and GLUT4 expression in skeletal muscle fibers. Therefore, lipid peroxidation ultimately causes insulin resistance. The adipose tissue that accumulates around the blood vessels (PVAT) secretes a variety of diffusible factors with vasomotor, vascular remodeling, pro- and anti-inflammatory, as well as pro- and anti-atherogenic effects. Adipose tissue also accumulates in the heart, both at the epicardial and pericardial levels (EAT and PAT, respectively). This cardiac adipose tissue secretes cytokines, chemokines, adipokines, growth factors, and FFAs, which indicate cardiomyocyte physiology, causing changes in heart function. In obese subjects, adipose tissue also accumulates in the liver and pancreas. In hepatocytes, the accumulation is intracellular, causing insulin resistance, in-creased hepatic lipogenesis, and the secretion of diffusible factors with effects on the hepatocytes themselves, promoting the functional changes described. In the pancreas, adipose tissue accumulates outside the pancreatic cells, and secretes chemokines and VEGF that promote macrophage infiltration into the pancreatic tissue. These macrophages secrete cytokines that interfere with insulin secretion. Finally, another characteristic of obese subjects is the accumulation of subcutaneous and visceral adipose tissue (SAT and VAT, respectively), preferably in the abdominal region. This adipose tissue secretes a multitude of diffusible factors, which exert effects on various body tissues and organs. Thus, in obese subjects, there is a crosstalk between adipose tissue and other body tissues and organs mediated by diffusible factors with local or generalized effects. For details, see text. Source for figure illustrations: https://scidraw.io/ (accessed on 8 September 2021).

In summary, there is wide crosstalk between visceral and subcutaneous adipose tissue through a set of diffusible factors secreted by this tissue that exert functional and pathophysiological changes in various tissues and systems, including cardiovascular, respiratory, gastrointestinal, renal, bone, and muscle systems. A schematic illustration of such crosstalk is shown in Figure 1.

### 2.5. Obesity: Fat Deposition, Dysfunctional Adipose Tissue, and Metabolic Complications

In the previous subsections, we described the accumulation of fat in various body tissues and organs in obesity conditions. The accumulation of fat changes between the different tissues and organs; thus, in skeletal muscles, the liver, and pancreas, there is an accumulation of fat in the form of lipid droplets or adipocytes in the parenchyma of these tissues or organs, while in the heart and skin, there is an excessive accumulation of fat outside the cells of these tissues. It is well known that some amount of adipose tissue surrounds several organs, and it has been shown to serve a physiological role [340]. Ectopic fat is defined by excess adipose tissue in locations not classically associated with adipose tissue storage, such as the omentum, liver, muscle, pancreas, heart, blood vessels, kidney, or neck, and is associated with the development of cardiovascular diseases [341,342]. Likewise, this excess fat in these regions, especially in the abdominal region, constitutes a sign of obesity [343], and the assessment of certain anthropometric parameters of visceral/abdominal obesity have been proposed as predictive parameters of cardiovascular risk [344]. In this context, it is not unreasonable to associate ectopic fat deposition with obesity, and the development of cardiovascular and metabolic complications.

It should also be noted that adipose tissue is deposited discreetly in the human body. Thus, the subcutaneous adipose tissue (SAT) stores more than 80% of the total fat in the human body, while the accumulation of intra- and retroperitoneal fat represents 10–20% of total body fat in men and 5–10% in women. SAT deports preferably subcutaneously in the abdominal, gluteal, and femoral regions. Intraperitoneal or visceral adipose tissues (VAT) are associated with digestive organs and include the omental (hangs off the stomach), the mesenteric (associated with the intestine), and epiploic (along the colon) [345]. As described above, SAT and VAT are composed by white adipose tissue (WAT), and their primary function is to maintain an energy homeostasis. WAT uptakes excess lipid and stores it in the form of triglycerides; it acts as an energy source and releases lipids in the form of non-esterified fatty acids when there is a demanded for energy. The maintenance of energy homeostasis depends not only on the balance between lipid uptake and release, but also on the sensitivity to stimulatory and inhibitory signals mediated by the sympathetic nervous system and hormonal system [346]. According to previous subsections, in obesity, WAT depots experience abnormal and excess expansion, either by increase in adipocyte number or adipocyte size. They become inflamed and release an increased amount of pro-inflammatory cytokines and other mediators, and a reduced amount of anti-inflammatory adiponectin and other adipokines into the circulation. With the development of obesity, adipose tissue becomes increasingly dysfunctional [346]. In addition, visceral obesity, including the accumulation of subcutaneous fat in the abdominal area, confers an increased risk for metabolic complications of obesity [347,348].

Metabolic syndrome (MetS), metabolically healthy obesity (MHO), and metabolically unhealthy obesity (MUO) are three phenotypes of metabolic complications of obesity. The term metabolic syndrome was first put forward by Reaven (1988) [349] to describe a cluster of interrelated cardiovascular and metabolic risk factors that predispose individuals to the development of type 2 diabetes mellitus and cardiovascular diseases. These well-known risk factors include abdominal obesity, hyperglycemia, hypertension, and atherogenic dyslipidemia. From a physiopathological point of view, the cardiometabolic risk factors that characterize MetS are usually accompanied by the presence of insulin resistance (IR), and by a systemic subclinical inflammation state secondary to the hyperactivity of innate immunity [350,351]. In the etiology of MetS, aging, genetics, inflammation, obesity, and sedentary lifestyle are recognized as predisposing factors. Although abdominal obesity is the major risk factor of the MetS that predispose to the metabolic and cardiovascular diseases [352], understanding by abdominal obesity, the accumulation of ectopic fat in the abdominal region, both viscerally and subcutaneously [343]. Visceral fat is a highly active tissue from the metabolic point of view. It is apparently more susceptible to lipolysis than subcutaneous adipose tissue and is associated with higher production of TNF-α, plasminogen activator inhibitor-1 (PAI-1), IL-6, IL-1β, monocyte chemoattractant protein-1 (MCP-1), macrophage migration inhibitory factor (MIF), C-reactive protein (CRP), chemokines from the CC and CXC families, IL-18, and IL-33, most of which are involved in insulin resistance. The size more than the number of visceral adipocytes is a key factor in abdominal obesity. Big visceral adipocytes are more prone to rupture, and cell rupture will obviously constitute a focus of inflammation, since macrophages infiltrate visceral adipose tissue to engulf cellular debris from dead adipocytes and contribute to the secretion of pro-inflammatory factors [353,354,355,356]. Visceral adipocytes are frequently subject to sudden pressure variations associated with cough, physical exercises, and sleep apnea. Intra-abdominal pressure is higher in obese patients, what may also impact adipocyte stability [357,358,359]. Despite this information, little is understood about the triggers of obesity-associated inflammation, but several mechanisms have been investigated in rodent models of dietary and genetic obesity. In this context, it has been observed that obesity gives rise to increased intestinal permeability, which results in higher circulating levels of LPS from intestinal Gram-positive bacterial species, and gut-derived LPS may initiate an inflammatory cascade via activation of pattern recognition TLR4 receptors in fat cells [360,361,362]. Mice subjected to intravascular infusion of free fatty acids causes an activation of NF-kappa-B in adipose tissue and the secretion of IL6 and MCP-1 by adipocytes and resident and/or infiltrated macrophages in adipose tissue during the course of infusion, and TLR4 is implicated in this response in macrophages and adipocytes to induce inflammatory signaling. Finally, in female C57BL/6J mice, TLR4 is in part required for the ability of high-fat diet to induce inflammatory mediators in peripheral tissues [360]. On the other hand, the proportion of M1 macrophages increases in liver tissue and adipose tissue of mice subjected to a high-fat diet that induces metabolic syndrome [363]. It is well known that M1-macrophages have a pro-inflammatory profile with the production of inflammatory cytokines and reactive oxygen species, but they are also ineffective macrophages in the elimination of dead adipocytes, or in the elimination of lipids [363].

Metabolically healthy obese (MHO) subjects are individuals with high fat mass, low ectopic fat, low triglycerides and inflammation, high HDL-cholesterol and adiponectin, and high insulin sensitivity. MHO are obese subjects with a favorable metabolic profile with the absence of metabolic complications including inflammation, dyslipidemia, and hypertension, and preserved insulin sensitivity despite excessive body fatness. The MHO phenotype is characterized by lower visceral adipose tissue content [364]. Higher insulin sensitivity in MHO subjects is accompanied with lower fasting glucose and insulin levels. MHO individuals present a favorable blood lipid profile as evidenced by lower triglycerides (TGs) and higher high-density lipoprotein (HDL) cholesterol levels, and favorable hepatic enzyme profile as evidence by lower aspartate aminotransferase (AST), alanine aminotransferase (ALT), and gamma-glutamyltransferase (GGT) levels [364]. Recently, a set of criteria to identify people with MHO has been proposed, based on (i) the absence of cardiometabolic disease, including absence of prediabetes, type 2 diabetes, dyslipidemia, nonalcoholic fatty liver disease, chronic kidney disease, cardiovascular disease, and absence of treatment with blood pressure, lipid or diabetic medications; (ii) a healthy cardiometabolic profile, such as fasting triglycerides <95 mg/dL, HDL-C ≥ 40 mg/dL in men and ≥50 mg/dL in women, fasting glucose <100 mg/dL and 2- hour OGTT glucose <140 mg/dL; (iii) normal blood pressure (<130/85 mmHg); (iv) normal intrahepatic triglycerides content (<5% of liver volume by imaging or <5% of hepatocytes with intracellular triglycerides by histology); and (v) normal insulin sensitivity (glucose infusion rate <8 mg/kg fate-free mass during an hyperinsulinemic-euglycemic clamp procedure at insulin infusion rate = 40 mU/m^2^/min [365].

Various underlying mechanisms have been proposed to explain the existence of MHO. There is growing evidence to suggest that subclinical inflammation may be the underlying mechanism that determines whether an individual is MHO. The subclinical inflammation profile includes low C-reactive protein (CRP) circulating levels, low levels of complement component 3 (C3), low levels of alpha necrosis tumor factor (TNF-a) and interleukin 6 (IL-6), and a low number of blood cells [366,367], but higher levels of pro-inflammatory adipokines, including leptin, resistin, IL-18, retinol-binding protein 4, lipocalin 2, angiopoietin-like protein 2, CC-chemokine ligand 2, and CXC-chemokine ligand 5 [367,368]. A second underlying mechanism is the expansion capacity of adipose tissue, based on when fat tissue needs to increase its storage capacity, can increase the size of adipocytes, or increase their number. These changes in adipose tissue are frequently accompanied by increased vascularization. In subjects in whom adipose tissue has difficulty expanding in the healthiest way, which is increasing cellularity, metabolic disease does appear. This happens in MHO subjects who maintain adequate storage capacity. MHO subjects have higher lipogenic and angiogenic capacities [367,369], and with adipocytes that have a lower average size and a favorable pattern of adipokine secretion [370]. On the other hand, MHO subjects are characterized by lower liver (intrahepatic) and visceral fat deposition, but higher leg fat content [371]. Accumulation of adipose tissue in the upper body (abdominal region) is associated with the development of obesity-related comorbidities (e.g., type 2 diabetes, cardiovascular and metabolic diseases, insulin resistance), whereas the accumulation of fat in the lower body (gluteofemoral region of leg) is associated with a protective lipid and glucose profile as well as a decrease of cardiovascular and metabolic diseases [372].

Metabolically unhealthy obesity (MUO) phenotype is characterized by high visceral fat volume with high liver fat content and low amount of leg fat accumulation, adipose tissue dysfunction, insulin resistance, and higher markers of inflammation [371,372,373]. MUO subjects have a similar risk of cardiovascular disease as MHO subjects, but a much higher risk of type 2 diabetes than MHO subjects [374]. MUO individuals display a pro-inflammatory adipose M1 macrophage profile characterized by the secretion of inflammatory cytokines including interleukin (IL)-6, IL-1b, and monocyte chemoattractant protein-1 (MCP-1). In addition, higher infiltration and recruitment of M1 macrophages has reported in adipose tissue of MUO subjects. Increased consumption of saturated fat, cholesterols, trans-fat, and fructose incites pro-inflammatory macrophage recruitment in MUO adipose tissue. Consumption of these dietary components in conjunction with dysfunctional adipogenesis results in adipocyte hypertrophy. This combined with decreased angiogenic signals, disrupted ECM turnover, and downstream pro-inflammatory cytokine secretion stimulates pro-inflammatory M1 macrophage recruitment. Systemic dysfunction in the form of a decreased vasoreactivity and oxidative stress concurrently fosters insulin resistance while promoting pro-inflammatory M1 macrophage recruitment into MUO adipose tissue. Once M1 macrophages enter the tissue, they secrete additional pro-inflammatory cytokines that recruit more M1 macrophages. Hypertrophic adipocytes in MUO adipose tissue communicate with the recruitment of M1 macrophages and secrete inflammatory leukotrienes (e.g., LTB4), which promotes insulin resistance in peripheral tissues and thus further recruitment of M1-proinflammatory macrophages. As a whole, it constitutes a vicious cycle of initiation and perpetuation of inflammation in the adipose tissue of MUO subjects [375]. In addition, MUO patients have a 44% decrease in capillary density and 58% lower VEGF signaling in the subcutaneous adipose, highlighting the occurrence of vascular rarefaction with hyperglycemia [376].

In summary, the previous evidences in both humans and animal models indicate that the quality of adipose tissue varies among these three metabolic disorders of obesity. Thus, in MetS and MUO subjects, abdominal obesity is observed, with secretion of inflammatory factors by adipose tissue, recruitment of M1 macrophages in adipose tissue, insulin resistance, and a high risk of type 2 diabetes and cardiovascular diseases. While the MHO subjects do not show abdominal obesity, but rather gluteofemoral fat accumulation, with a low to zero risk of type 2 diabetes and cardiovascular diseases, higher insulin sensitivity, and low or very low secretion of inflammatory factors by adipose tissue.

## 3. Obesity and Pain: Crosstalk between Adipose Tissue and Nociceptive Somatosensory Nervous System

Low back pain [46,47,51,377,378,379,380], foot pain [72,381,382], knee pain [69,383,384], hip pain [71,385], shoulder pain [386], musculoskeletal pain [60,387,388,389], headache/migraine [74,76,390,391,392,393,394], neuropathic pain caused by nerve injuries and diabetic neuropathy [395,396,397,398], and fibromyalgia [399,400], were reported in overweight/obese subjects. These findings suggest that overweight/obesity, which, as mentioned before, is characterized by excess accumulation of adipose tissue, may trigger pathological pain development, both neuropathic and nociplastic.

Distal symmetric polyneuropathy (DSP), a type of peripheral neuropathy, has been described in subjects with metabolic syndrome [401,402]. It is well known that DSP is associated with pain, falls, and reduced quality of life [403,404,405,406,407,408,409,410,411,412]. On the other hand, metabolic syndrome also is associated with an elevated risk for cryptogenic sensory peripheral neuropathy (CSPN), which preferentially affects small unmyelinated axons early in its course, and it may affect autonomic and large fibers [413]. CSPN is usually diagnosed on the basis of pain, numbness, and/or tingling in the distal extremities without symptoms of weakness [414,415]. On the other hand, it was reported that patients with neuropathic pain, the pain intensity in the overweight group was significantly higher than that in the normal-weight group [397]. However, using noxious heat and cold stimuli across multiple body locations and durations of stimulation, no differences in suprathreshold pain intensity or unpleasantness ratings between obese and healthy groups were seen. These results suggest that increased adiposity does not in-and-of-itself alter nociception [416]. It should be noted that, in the scientific literature, there are several excellent reviews on the relationship between obesity and pain in humans. In summary, the scientific evidence reviewed in these publications indicates that obesity is significantly associated with the presence and extent of pain complaints, and the prevalence of pathological pain (e.g., low back pain, osteoarthritis, fibromyalgia) is higher in obese or morbidly obese subjects compared to subjects with normal weight. Longitudinal studies suggest that obesity may be a risk factor for developing chronic pain. Obesity also increases the risk factor for developing headache. Several studies showed that obese people exhibited decreased pain threshold to electrical and mechanical, whereas other studies also show inconsistent results across body parts, suggesting that the reduced pain threshold may not be present for all body areas in obesity. The increased loading due to heavy weight on joints and the spine is one of the most discussed links between obesity and pain. Another link between obesity and pain is adipose tissue. In this sense, obesity may be characterized by a low-grade chronic inflammatory state as reflected by elevated levels in many inflammatory markers in the serum, some of which may act as algogenic substances by stimulating visceral nociceptors or sensitizing nociceptive neurons in the central nervous system. In addition, overweight/obese individuals have an increased risk of various metabolic disorders, thus they have increased vulnerability to the neuropathic disorders associated with conditions, such as diabetes, and some of these peripheral neuropathies present with pain [382,417,418,419,420,421,422,423,424,425,426,427,428,429,430,431]. On the other hand, several studies were reported linking metabolic diseases of obesity with pain. In this context, MetS was associated with neck pain. This association was stronger in males, but the prevalence of neck pain was higher in females [432]. MetS was also significantly associated with low back pain among women only [433,434]. About 40% patients with chronic low backache have metabolic syndrome, and they have more severe pain and disability [435]. Chronic pain due to osteoarthritis in both knees were reported in patients with metabolically healthy obesity [436]. In addition, it was also reported that over the course of two decades, decline in physical functioning and worsening of bodily pain among initially healthy obese adults was two- and six-times greater than among initially healthy normal-weight adults, respectively. These changes occurred at similar rates for both healthy and unhealthy obese adults [437].

According to the International Association for the Study of Pain (IASP), the definition for neuropathic pain is “the pain caused by a lesion or disease of the somatosensory nervous system”, whereas nociplastic pain is “the pain that arises from altered nociception despite no clear evidence of actual or threatened tissue damage causing the activation of peripheral nociceptors or evidence for disease or lesion of the somatosensory system causing the pain” [438].

Neuropathic pain includes: (i) trigeminal neuralgia; (ii) peripheral nerve injury by physical trauma; (iii) painful polyneuropathy associated with polyneuropathies caused by metabolic, autoimmune, familial or infectious diseases, exposure to environmental or occupational toxins, or treatment with a neurotoxic drug; (iv) postherpetic neuralgia defined as pain persisting for ≥ 3 months following the onset or healing of herpes zoster; (v) painful radiculopathy defined as pain caused by a lesion or disease involving the cervical, thoracic, lumbar or sacral nerve roots; (vi) neuropathic pain associated with spinal cord injury defined as pain caused by a lesion or disease of the somatosensory pathways in the spinal cord; (vii) neuropathic pain associated with brain injury defined as pain caused by a lesion or disease of the somatosensory cortex, connected brain regions, or associated pathways in the brain; (viii) post-stroke pain defined as pain caused by a cerebrovascular lesion, infarct or hemorrhage, of the brain or brainstem; and (ix) neuropathic pain caused by multiple sclerosis in the somatosensory brain regions or their connecting pathways [439]. On the other hand, under the definition of nociplastic pain are included nonspecific back pain, nonspecific peripheral joint pain, fibromyalgia, complex regional pain syndrome (CRPS) type 1, and visceral pain disorders (e.g., irritable bowel syndrome, bladder pain syndrome) [440,441,442,443].

Together, with the scientific evidence, it can be affirmed that there is a solid rationale in the implication of obesity and its metabolic complications in pathological pain in humans. Given what is stated in the previous paragraphs, both neuropathic pain and nociplastic pain are found in either obese or overweight subjects. In this context, a large number of diffusible factors synthesized and released by the different adipose tissues in obese subjects (Table 1) have been described, and some were shown to be able to interact with nociceptive nerve fibers, favoring the appearance of pain. This interaction may be explained by the fact that nociceptive nerve fibers are located around body tissues and organs. In the heart, calcitonin gene-related peptide (CGRP)-positive nerve fibers, identified as nociceptive afferent fibers, are shown as free endings in the epicardium of mice [444]. Epicardial afferent nociceptive nerve fibers also were reported in the dog’s heart [445], and in the epicardium of the heart of the cat [446]. Human epicardium also shows nerve terminals immunoreactive to either CGRP or substance P (SP), identified as afferent nociceptive fibers [447]. Moreover, peptidergic fibers (e.g., positive for CGRP and/or SP) have been visualized in the pericardium of chicken and human hearts [448,449]. As for the digestive system, CGRP- and SP-positive afferent nociceptive fibers are observed in the head of the mouse pancreas [450] and rat liver shows afferent nociceptive neurons sensitive to capsaicin administration [451]. Moreover, in resected human bowel tissues, nociceptive afferent nerve fibers more sensitive to algogenic mediators were identified [452]. In mice, mechanical distension and stretch-not heat, cutting, or pinching-reliably evoke pain from distal colon and rectum, suggesting that the colon wall showed nociceptive afferent fibers that respond to these stimuli [453]. Regarding respiratory system, in guinea pig, it has reported that nociceptive C-fiber population innervating the lungs [454], TRPV1- and TRPV2-immunoreactive nociceptive afferent nerve ending were reported in rat trachea [455], and TRPA1 nociceptive afferent fibers have been reported in mouse lungs [456]. Moreover, nociceptive afferent nerve fibers are observed in the parietal peritoneum of the rat [457] and it was reported that several drugs produce an antinociceptive effect in the mouse peritoneum by interacting with sensory nerve endings, suggesting that peritoneum may be innervating by nociceptive fibers [458]. Moreover, in the mice, most group IV muscle afferents are chemosensitive rather than mechanically or thermally sensitive, and the majority of these C fibers are muscle polymodal nociceptors [459]. Regarding skeletal muscle systems, in rats, muscle fascia shows a rich fiber network of thin-fiber receptors (Aδ- and C-fibers) [460]. In human skeletal muscle has been observed positive CGRP and SP afferent nociceptive fibers associates with myocytes [461]. The afferent nociceptive nerve fibers located in human muscle respond to mechanical and chemical stimuli [462]. Finally, focusing on skin, in human biopsies, researchers reported nociceptive intraepidermal nerve fibers [463,464,465] and rat and mice skin biopsies revealed intraepidermal free nerve fiber endings identified as afferent nociceptive fibers [466,467,468,469,470]. These evidences indicates that nociceptors or nociceptive afferent fibers are located in most body tissues and organs and, therefore, the substances released by adipose tissue that accumulate in these tissues and organs in obese subjects can interact with the nociceptors, causing their depolarization or their sensitization; thus, developing pain signals.

The adipose tissue is composed of various types of cells, such as adipocytes (e.g., progenitor and mature cells), endothelial cells of the wide vascular network that supplies adipose tissue, fibroblast, and immune cells (e.g., natural killer, T-cells, lymphocytes, neutrophils, macrophages, dendritic cells) [345,471,472]. The different cellular elements that make up adipose tissue are involved in the synthesis and release of various factors secreted by the adipose tissue accumulated in body organs and tissues, including adipokines, cytokines, chemokines, growth factors, and other diffusible factors. All of them may participate in pathological pain development by interacting with muscle, and skin and visceral nociceptors.

### 3.1. Adipokines

Adipokines are synthesized and released mainly by adipocytes, the main cells of adipose tissue that accumulate in tissues and organs. In an experimental model of inflammatory pain by intraplantar carrageenan injection, the intrathecal adiponectin administration attenuates both thermal and mechanical hyperalgesia whereas intraplantar administration alleviates only thermal hyperalgesia [473]. These findings suggest that adiponectin secreted by SAT may interact with their receptors located in skin afferents nociceptive fibers alleviating thermal hyperalgesia. Another possibility is that adiponectin interacts with AdipoR1/R2 receptors located in macrophages and mast cells that have been activated by carrageenan, inducing in them a lower release of pro-inflammatory factors, which interact with cutaneous nociceptors. Actually, it is well known that macrophages express AdipoR1/R2 and adiponectin reduces the production of pro-inflammatory cytokines by the activated macrophages that showed a M1 phenotype [474]. Mast cells also express AdipoR1/R2 receptors, and adiponectin causes a significant reduction of pro-inflammatory factors and histamine release [475]. These findings indicate that analgesic effect of adiponectin is mediated by regulation of inflammatory factors, secreted by macrophages and mast cells, which interact with the afferent nociceptive fibers in the skin.

On the other hand, intraplantar injection of capsaicin in Sprague–Dawley rats causes an overexpression of adrenomedullin (ADM) in CGRP- and IB4-positive dorsal root ganglion neurons identified as nociceptors. Capsaicin induces a release of ADM by these nociceptors, which promotes thermal hyperalgesia. Moreover, intrathecal administration of ADM causes thermal hyperalgesia and histological results have been revealed that CGRP- and IB4-positive afferent nociceptive fibers express receptors for ADM (e.g., CLR, RAMP2, and RAMP3) [476]. These results suggest that inflammation of skin causes release of ADM by nociceptors that in turn stimulates the nociceptors, playing a role similar to CGRP. Consequently, ADM is a pro-nociceptive molecule secreted by afferent nociceptive fibers, but also the ADM secreted by SAT may interact with their receptors located in skin nociceptors causing pain.

Regarding angiotensin-II (Ang-II), its injection into mouse hind paws induces mechanical but not thermal hyperalgesia and its intrathecal injection did not induce mechanical allodynia. It is known that Ang II-induced mechanical pain sensitivity operates exclusively via AT2R at a peripheral level, not spinally. In addition, Ang-II induces peripheral macrophages infiltration in mouse hind paw skin, which also respond to Ang-II by secreting ROS, since these cells also express the receptor for angiotensin-II (AT2R) [477]. According to these results, Ang-II may be considered as a pro-nociceptive molecule secreted by subcutaneous and perivascular adipose tissues, causing pain via binding with AT2R located in afferents nociceptive fibers.

As for apelin, peripheral injection of a single dose causes thermal hyperalgesia [478], but its intrathecal administration alleviates CFA-induced thermal and mechanical hypersensitivity in mice [479]. In models of formalin-induced pathological pain, intravenous injection of apelin reduces pain response during the second phase of the mouse formalin test, whereas intramuscular injection of apelin reduces licking/biting time during the second phase only at higher dose. [480]. In contrast, after chronic constriction injury (CCI) of the sciatic nerve, intrathecal administration of apelin does not alleviate thermal hyperalgesia and mechanical allodynia [481]. These results suggest that the peripheral administration of apelin is pronociceptive and its central administration exerts analgesic effects in inflammatory pain but not in neuropathic pain. Hence, the local release of apelin by the accumulation of subcutaneous adipose tissue may trigger pain.

With regard to chemerin, its intrathecal administration alleviates both thermal and mechanical hypersensitivity in mice after CCI [482]. In Complete Freund’s adjuvant (CFA, 0.5 mg/mL saline) experimental model of inflammatory pain, chemerin may also exert anti-nociceptive effects. Spinal cord slices from animals subjected to CFA-injection were treated with chemerin while whole-cell patch-clamp recordings are made in spinal lamina I neurons. Under these conditions, when capsaicin was applied, an increase in the firing frequency of excitatory potentials was observed in spinal neurons, which were attenuated by chemerin. At the central level, chemerin reduces the hyperexcitability of spinal neurons, exerting an anti-nociceptive effect [483]. In addition, intrathecal administration of chemerin alleviates hyperalgesia in CFA-induced hind-paw inflammation in rats [484]. All these findings suggest that chemerin may exert anti-nociceptive effects on both inflammatory and neuropathic pain, exerting its effects at spinal cord level.

Another molecule playing roles in adipose tissue and nociceptive somatosensory nervous system crosstalking is the leptin. In obese mice, which show higher levels of circulating leptin, formalin-induced nociceptive responses (flinching behavior) were significantly reduced. These results suggest that leptin may attenuate pain associated with inflammation [485]. In contrast, it was reported that leptin derived from adipocytes in the epineurium of the sciatic nerve, contributes to the development of tactile allodynia through the production of the molecular substrates for neuropathic pain (e.g., overexpression of mRNA for MMP-9, COX-2, and iNOS) by stimulation of the JAK-STAT pathway in macrophages. MMP-9 and iNOS mRNA levels increased in a macrophage cell line treated with leptin. Hence, these findings suggest that leptin in the PNS is a novel mediator of tactile allodynia induced by peripheral nerve injury [486]. On the other hand, intrathecal administration of leptin alleviates both thermal hyperalgesia and mechanical allodynia and reduces the expression of IL-6 and TNFα in Sprague–Dawley rats after CCI, an experimental model of neuropathic pain [487], these results suggest that circulating leptin reduces the expression of pro-inflammatory cytokines in neuropathic and inflammatory animal models of pain, alleviating hyperalgesia in these animal models of pain. However, local release of leptin in injured nerve promotes overexpression of inflammatory mediators that improve hyperalgesia. Consequently, the exact role of leptin on the development of hyperalgesia is still controversial.

There are no experimental studies on the effect of omentin administration on either neuropathic or nociplastic pain. However, it was shown that circulating levels of omentin decrease in patients with temporomandibular pain [488]. On the other hand, it was reported that osteopontin levels are elevated in patients with osteoarthritis, a degenerative joint disease characterized by loss of articular cartilage, inflammation, and pain [489]. In addition, osteopontin levels in synovial fluid is associated with the severity of joint pain and cartilage degradation after anterior cruciate ligament rupture [490]. In mice subjected to spared nerve injury (SNI), mechanical and thermal withdrawal thresholds tested in intact adult wild type and osteopontin knockout animals showed that the mechanical allodynia was attenuated in osteopontin-KO mice respect to wild type mice. In contrast, no differences in thermal latencies (thermal hyperalgesia) were observed between the two genotypes [491]. These results suggest that osteopontin expression after SNI, a model of neuropathic pain, may facilitate mechanical but not thermal hyperalgesia. Clinical studies also suggest that increased expression of osteopontin increases pain sensation. In relation to osteoprotegerin (OPG), pre-clinical studies indicate that it reduces pain behavior development and joint pathology in a model of osteoarthritis [492], and alleviates pain in rats subjected to bone cancer [493,494]. On the other hand, serum resistin increases in subjects with bilateral knee osteoarthritis [495], and in patients with hip and knee osteoarthritis [496]. However, no pre-clinical studies have been performed on the effect of the application of resistin in neuropathic and nociplastic pain. As for vaspin, it was reported a significant association between vaspin circulating levels and low back pain [497], but not pre-clinical studies, have been performed with the aim of studying the effect of vaspin on neuropathic or nociplastic pain. Finally, there are no pre-clinical studies on the role of visfatin in pathological pain or its development. It is known that visfatin levels increases in patients with osteoarthritis, but this increase could not be related to the pain indicated by the patients [498].

### 3.2. Cytokines and Chemokines

Cytokines and chemokines are mainly synthesized and released by immune cells that are part of the adipose tissue accumulated in body tissues and organs. Growing evidences suggest that both cytokines and chemokines are involved in sensitization of nociceptors, that is, in peripheral sensitization, and in the depolarization of nociceptive primary afferent fibers. For instance, peripheral administration of TNF-alpha [499,500], IL6 [501] and IL1 [502,503,504] cause excitability and/or sensitization of C- and A-delta-nerve fibers. It should be noted that cytokines do not always trigger excitability of nociceptive afferent fibers, thus IL6 causes hypoactivity of nociceptive fibers [505]. On the other hand, in IL17-KO mice it has reported less behavioral hypersensitivity after partial sciatic nerve ligation and a decrease in inflammatory cell infiltration and pro-inflammatory cytokine levels in injured nerves [506]. Intraperitoneal administration of RANTES also attenuates hypersensitivity after partial sciatic nerve ligation. In addition, macrophage infiltration and pro-inflammatory cytokine levels also decreased in injured nerve [507]. These findings suggest that IL17 and RANTES participate in recruitment of inflammatory cells to injury sites releasing inflammatory mediators that causes peripheral sensitization. Consequently, indirectly both IL17 and RANTES participate in the peripheral sensitization of injured nociceptive afferents nerve fibers. Likewise, it was reported that the plantar administration of IL17 causes acute hyperalgesia indirectly by inducing TNF from resident cells [508]. Respect to IL-33, this cytokine contributes to peripheral and spinal cord nociceptor neuron sensitization in innate and adaptive inflammatory immune responses as well as in neuropathic and cancer pain [509].

As for the chemokines, it is known that, for example, the administration of MCP1/CCL2 causes hyperexcitability of C-fibers [510]. Actually, it was reported an upregulation of TRPV1 and Na (v)1.8 channels in DRG nociceptive neurons treated with CCL2, suggesting that this chemokine facilitate pain transmission [511]. In the same context, mechanical hyperalgesia has been evidenced in animals subjected to lysophosphatidylcholine injection that causes sciatic nerve demyelination. This pain response is associated with an upregulation of CCR2, CXCR4, CXCR3, and CCR5 receptors in dorsal root ganglia neurons, and MCP1/CCL2 expression in IB4-positive neurons [512]. These finding suggest that upregulation of chemokines and their receptors contributes to sensitization of peripheral nociceptors. In the same line, CCL5-deficient mice showed less behavioral hypersensitivity and a decrease of both macrophage infiltration and pro-inflammatory cytokines in injured areas after partial sciatic nerve ligation, suggesting that CCL5 participates in the peripheral sensitization [513]. Finally, CXCL1 is a chemokine that promote both nociceptor and central sensitization via its main receptor CXCR2 [514] and it is known that chronic morphine administration enhances incision-induced nociceptive sensitization in mice mediated by the upregulation of peripheral CXCL1/CXCR2 expression [515]. It is worth mentioning that the administration of fractalkine into the DRG (L5) produces mechanical hyperalgesia, causing an activation of satellite glial cells leading to the production of TNFα, IL-1β, and prostanoids, which are likely responsible for the maintenance of inflammatory pain [516].

### 3.3. Growth Factors and Other Diffusible Factors

Fibroblasts and endothelial cells in adipose tissue can synthesize and secrete growth factors and other diffusible factors that, upon reaching skin, muscle, and visceral nociceptors may trigger pain behavior. It has been evidenced that basic fibroblast growth factor (bFGF) increases the NaV1.8 current density in cultured adult rat DRG neurons. In addition, bFGF sensitizes DRG neurons at the cellular level by induction of Erk1/2 phosphorylation and by an Erk1/2-dependent increase of the NaV1.8 current density. Finally, it is known that intradermal injection of bFGF in rats causes mechanical hyperalgesia [517]. As for transforming growth factor-β1 (TGF-β1), it potentiates ASIC-mediated electrophysiological activity and nociceptive behaviors in rats [518]. Moreover, rats with intratibial inoculation of carcinoma showed thermal hyperalgesia and high levels of TGF-β1. TGF-β1-mediates bone cancer pain by sensitization of TRPV1 in primary sensory neurons [519]. On the other hand, in an experimental model of pancreatitis, it was reported that intrathecal infusion of TGF-beta causes hyperexcitability of nociceptive pancreas nerve fibers, and contributes to pancreatic pain hypersensitivity [520]. After partial sciatic nerve ligation (PSL), mechanical and thermal hyperalgesia are observed in injured rats, and mRNA VEGF levels increase in injured sciatic nerve that is expressed in infiltrated neutrophils and macrophages. In addition, VEGF receptors are expressed in endothelial cells and macrophages, and angiogenesis is observed in the injured sciatic nerve. Taken together, VEGF is upregulated in injured peripheral nerves and participates in angiogenesis and prolonged pain behaviors through its receptors [521]. After partial saphenous nerve injury, the exogenous administration of two isoforms of VEGF has opposing effects: VEGF-A165a exacerbates, whereas VEGF-A165b alleviates thermal and mechanical hyperalgesia [522].

Regarding other diffusible factors, accumulating evidence suggests that reactive oxygen species (ROS) contributes to the sensitization during persistent pain [523]. Spared nerve injury (SNI) causes an increase of ROS in mice. NOX enzymes are important ROS sources in somatic cells, and Nox4 expression was seen in IB4-positive dorsal root ganglia cells. These findings suggest that Nox4 is implicated in the production of ROS after SNI causing hyperalgesia [524]. Nitric oxide directly evokes pain in humans when it is injected into the skin [525]. Intraplantar and intrathecal administration of sodium hydrosulfide (NaHS), a donor of H2S, also causes hyperalgesia in rats [526,527].

**Figure 2:**
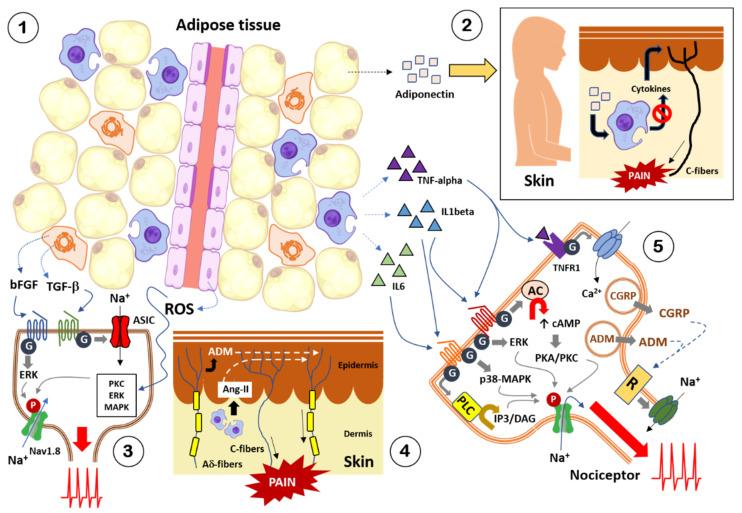
Crosstalk between adipose tissue, skin, and visceral nociceptors. There is a multitude of diffusible factors secreted by adipose tissue that interact with nociceptors, triggering pain. (1) Adipose tissue is made up of an accumulation of adipocytes, macrophages, lymphocytes, neutrophils, T-cells, natural killer cells, and fibroblasts, as well as an extensive network of blood vessels. (2) Adipocytes secrete adipokines, such as adiponectin, which can reach the skin through the bloodstream. Under an inflammatory condition of the skin, the cutaneous macrophages secrete cytokines, which excite and sensitize the cutaneous nociceptors, triggering pain. Adiponectin acts on these macrophages, inhibiting the release of pain-inducing cytokines. Therefore, this adipokine has analgesic effects. (3) Fibroblasts from adipose tissue accumulated in body tissues and organs secrete growth factors that interact with G protein-coupled receptors on the membrane of nociceptors. Some of these growth factors activate intracellular cascades (e.g., ERK) that allow peripheral sensitization through ion channel phosphorylation (e.g., Nav1.8), thus favoring cation fluxes that trigger depolarization of nociceptors and pain. Other growth factors modulate ion channel opening (e.g., ASIC) and cation entry and depolarization of the nociceptor. Together, growth factors secreted by fibroblasts in adipose tissue cause hyperexcitability of nociceptors and pain. On the other hand, the endothelial cells of the blood vessels that supply adipose tissue secrete oxygen free radicals (ROS) that diffuse to the nociceptors, modulating various intracellular signaling pathways, which cause ion channel phosphorylation, peripheral sensitization and hyperarousal of nociceptors. (4) Inflammation of body tissues, such as the skin, triggers the nociceptors themselves to release adrenomedullin (ADM) or macrophages to release angiotensin-II (Ang-II), two adipokines also secreted by adipose tissue. Both diffusible factors act on receptors in the membrane of cutaneous (or visceral) nociceptors, modulating the opening of ion channels, cation fluxes, and depolarization, causing pain. (5) The macrophages (and other immune cells) of the adipose tissue accumulated in the organs of obese subjects, secrete various cytokines, which by diffusing can interact with specific receptors present in the membrane of visceral nociceptors. The interaction of cytokines is carried out on receptors coupled to G proteins. Some of these interactions (e.g., TNF-alpha) modulate the opening/closing of ion channels, causing variations in the influx of calcium ions. These ions favor the fusion of vesicles loaded with peptides (e.g., pep-tide related to the calcitonin gene or CGRP, adrenomedullin or ADM) present at the terminals of nociceptors. Thus, adipose tissue cytokines cause the release of CGRP and ADM by visceral nociceptors, peptides that interact with their respective receptors (R) that end up regulating the opening of ion channels that favor the entry of sodium ions and depolarization of the nociceptor. At other times, cytokines released by adipose tissue regulate various intracellular cascades (e.g., cAMP/PKA-PKC, ERK, p38-MAPK, IP3/DAG) responsible for the phosphorylation of ion channels and other metabotropic receptors on the nociceptor membrane, triggering peripheral sensitization and hyperexcitability of the nociceptive afferent nerve fiber. In summary, in obese subjects, there is a powerful crosstalk between the accumulated adipose tissue in the body organs and the nociceptive afferent nerve fibers present in these organs, so that the diffusible factors secreted by the adipose tissue cause sensitization and hyperexcitability of the nociceptor, triggering pain. For details, see the main text. Source for figure illustrations: https://scidraw.io/ (accessed on 8 September 2021).

In this section, evidence has been presented that neuropathic and nociplastic pain are symptoms and signs associated with obese subjects, and that adipose tissue, skin, heart, pancreas, liver, and other body organs of different mammalian species, including humans, present nociceptors and, therefore, can be excited and/or sensitized by the chemical substances released by the adipose tissue that is deposited on them in obese subjects. A schematic illustration of such processes involving adipose tissue chemical substance release and nociceptor activation is shown in Figure 2.

## 4. Effect of Physical Exercise on Adipose Tissue and Other Body Tissues, Organs, and Systems in Obese Subjects and Its Impact on Pathological Pain

The reduction of adipose tissue is one of the ways to reduce weight in obese subjects, and this can be achieved through changes in diet and modifying energy expenditure through the prescription of physical exercise. In this sense, increasing energy expenditure can help reduce excess adipose tissue in an obese subject [528]. Guidelines by the American College of Sports Medicine (ACSM) include either aerobic or anaerobic exercise. Aerobic exercise (e.g., running, cycling, rowing, etc.) is an exercise that exhausts the oxygen in the muscles, but oxygen consumption is sufficient to supply the energy demands placed on the muscles and does not need to derive energy from another source, whereas anaerobic exercise (or resistance exercise, e.g., weight lifting) is oxygen consumption that is not sufficient to supply the energy demands placed on the muscles, and muscles must break down other energy supplies, such as sugars, to produce energy and lactic acid [529].

International guidelines recommend people living with obesity should be prescribed a minimum of 300 min of moderately intense activity per week for weight loss [529,530]. However, the most efficacious exercise prescription to improve anthropometry, cardiorespiratory fitness (CRF) and metabolic health in this population remains unknown. Despite this, in a recent meta-analysis study, it was reported that while any type of exercise intervention is more effective than control, weight loss induced is modest. Interventions that combine high-intensity aerobic and high load resistance training exert beneficial effects that are superior to any other exercise modality at decreasing abdominal adiposity, improving lean body mass and increasing cardiorespiratory fitness [531]. Another systematical review and meta-analysis study revealed that low-volume high-intensity interval training (HIIT) is inefficient for the modulation of total body fat mass or total body fat percentage in comparison with a non-exercise control and moderate-intensity continuous training (MICT), in overweight and obese adults. In addition, HIIT does not produce a significant change in lean body mass compared to a non-exercising control. However, low-volume HIIT induces greater improvements in cardiorespiratory fitness than a non-exercising control and MICT in normal weight, overweight, and obese adults. Low-volume HIIT, therefore, appears to be a time-efficient treatment for increasing fitness, but not for the improvement of body composition [532]. High-intensity interval training (HIIT) is characterized by short bouts of high-intensity submaximal exercise interspersed with rest periods. Low-volume HIIT, typically involving less than 15 min of high-intensity exercise per session [533].

All of these studies suggest that physical exercise together with the establishment of a diet low in calories may allow body weight to decrease by reducing adipose tissue deposits and increasing muscle mass in obese subjects. This section will review the effect of physical exercise on adipose tissue and other body tissues and organs, mainly in obese but in non-obese subjects, and the impact of such exercise on pain behavior. Here, both studies carried out in humans and in animal models have been included.

Growing evidence suggest that physical exercise interventions are generally effective at reducing body mass. Aerobic training and combination of aerobic and resistant training, reduce both total body mass and fat mass, more than only resistance training. However, resistance training and combination of aerobic and resistance training increase lean body mass, more than aerobic training. These findings suggest that aerobic training may be the optimal mode of exercise for reducing fat mass and body mass, but a program including resistance training is needed for increasing lean mass in overweight/obese individuals [38]. In addition, combination of aerobic and resistant training improves cardio-respiratory parameters (e.g., maximal oxygen consumption or VO2max) and reduces fat percentage and abdominal fat percentage in overweight and obese individuals compared to the lack of exercise [534]. In obese subjects, an exercise training program consisting of aerobic exercise and resistance exercise, with adjustment of exercise intensity every 4 weeks, it was reported to increase maximal oxygen consumption and maximal power output, and to improve peripheral insulin sensitivity but not hepatic and adipose tissue insulin sensitivity. Moreover, total fat mass and body fat percentage slightly decrease but body weight, body mass index, fat free mass, and plasma glucose, non-esterified fatty acid and triacylglycerol concentrations remained unaltered after this training intervention [535]. On the other hand, resistance or aerobic exercise training in sedentary and overweight subjects cause a reduction of C-reactive protein (CRP) but not IL6 [536]. Recently, it was reported that aerobic training, resistance training, and combined training lead to SAT reduction, but aerobic exercise was shown to produce greater efficacy in decreasing SAT [40].

In rats, resistance training induces a significant reduction of body weight and subcutaneous and epididymal white adipose tissue, but the brown adipose tissue is not affected by training. After training, animals showed smaller adipocytes [537].

Aerobic training increase angiogenesis in skeletal muscle but not in subcutaneous adipose tissue (SAT) in insulin-resistant subjects [538], but in obese subjects this training pattern, elevates VGEF expression in SAT and promotes angiogenesis in this adipose tissue [539]. In overweight/obese subjects, aerobic training: (i) reduces the percentage of fat mass without changing the body weight, and modifies adipose tissue lipolysis through an enhancement of beta-adrenergic response [540]; (ii) increases circulating and adipose tissue adiponectin levels [541]; (iii) facilitates a reduction of hepatic fat content without significant changes in weight and visceral fat [542], causing a greater reduction in intra-abdominal adipose tissue than diet [44]. Moreover, after strength and endurance training intervention, insulin sensitivity, VO2max, strength, whole-body, muscle fat content, and abdominal adipose depots were improved in overweight subjects, and hepatic fat, waist circumference, and subcutaneous adipose tissue were reduced in overweight training subjects. Visceral fat was preferentially lost compared with other adipose depots [543]. In addition, endurance-strength training reduces hypertension and decreases fat mass and visceral adipose tissue and increases free fat mass, appendicular lean mass index, and lean mass index [544].

In obese mice, strength and endurance training causes a decrease of whole-body insulin resistance, improve glucose tolerance, and higher activation of the insulin pathway in skeletal muscle. In addition, training increase citrate synthase (CS) activity in skeletal muscle, but not in adipose tissue. These results suggest that the improvement in insulin resistance induced by training is related to mitochondrial adaptation in skeletal muscle, but not in adipose tissue [545].

Combination of resistance and endurance training in overweight subjects cause a reduction of weight, liver fat content and plasma fetuin-A levels. Exercise intervention improve slightly free fatty acids (FFAs) concentration but significantly glucose infusion rate (GIR). These finding suggest that long-term exercise may increase insulin sensitivity by lowering plasma concentration levels of the hepatokine fetuin-A, probably by interfering with adipose tissue [546]. This training pattern also causes a reduction of VAT and IL6 and TNF-alpha in blood plasma, whereas improve insulin sensitivity in humans [547].

Endurance training causes that fat cell weight and lipoprotein lipase activity were lower in abdominal adipose tissue of trained than sedentary subjects. Epinephrine and isoproterenol stimulated lipolytic responses and beta-adrenergic sensitivity of abdominal adipocytes were higher in trained than sedentary subjects, whereas alpha-adrenergic sensitivity was lower in endurance-trained than sedentary subjects. These findings indicate that trained subjects are characterized by a preferential lipid mobilization from the abdominal fat deport, and a reduction of lipoprotein lipase activity with changes in the lipolytic cascades [548]. This training pattern also causes: (i) a reduction of weight body and fat cell size [549]; (ii) a reduction of abdominal fat mass associated with improve of hepatic insulin sensitivity and peripheral insulin sensitivity related with endurance training only [550]; (iii) a decrease of fat weight cell and increased adipocyte epinephrine maximal stimulated lipolysis [551]; (iv) reduction of insulin resistance with increased plasma levels of adiponectin [552]; (v) an increase of blood high-density lipid cholesterol content, maximal exercise capacity, ventilatory threshold, and muscle performance in trained subjects [553]; (vi) a reduction of total cholesterol, triglycerides, and low-density lipoprotein cholesterol (LDL-C), and an increase of high-density lipoprotein cholesterol proteins [554,555]; (vii) reduction of tissue plasminogen activator antigen levels (a cardiovascular risk marker) in overweight subjects [556]; (viii) an improve of maximal oxygen uptake [557].

Strength training improves insulin sensitivity in subcutaneous adipose tissue of obese subjects [558], without altering plasma levels of adipokines (e.g., adiponectin, leptin) [559]. Resistance training in combination with proper diet promotes a reduction of body fat in overweight older adults [560], and this intervention (exercise and diet) improves the cardiovascular conditions of the older subjects [561]. As for pubertal children, resistance training is effective for subjects with low adiposity, but it is ineffective for obese pubertal subjects [562]. Finally, abdominal resistance training, besides diet, did not reduce abdominal subcutaneous fat thickness compared to diet alone in overweight/obese women [563].

Hence, previous experimental evidence in animals and humans shows that different patterns of physical training exert variable effects on obese subjects’ adipose tissue, but most of these studies show changes that lead to improvements in adiposity. It should be noted that part of the heterogeneity of the results of these studies may be due to a diversity of ways of executing physical exercise (e.g., treadmill, swimming, wheel running, etc.), as well as the protocols used on intensity and duration of the physical exercise.

**Figure 3:**
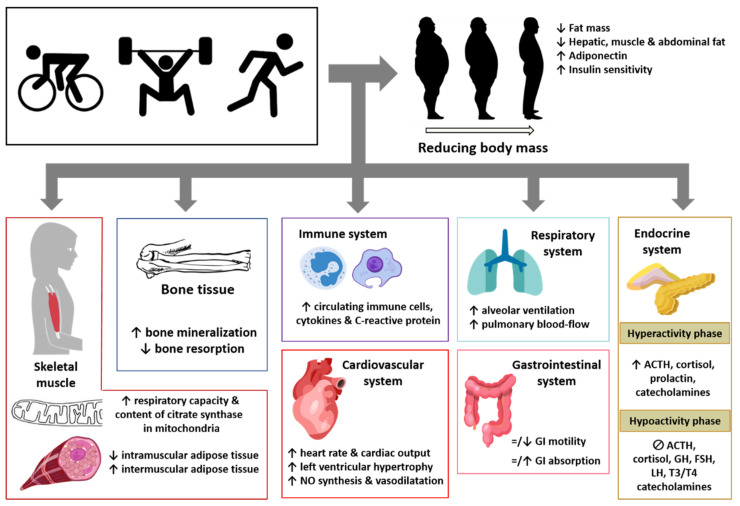
Overall vision of the effect of physical exercise on body tissues and systems. Physical exercise causes a reduction in body mass with a direct impact on the reduction of accumulated fat, which facilitates an increase in the secretion of adipokines that favor tissue insulin sensitivity in obese subjects. Likewise, physical exercise also has a direct impact on various organs and body systems. On skeletal muscle, it causes metabolic changes especially in mitochondrial physiology, increasing respiratory capacity and the content of Krebs cycle enzymes (e.g., citrate synthase). It also causes a decrease in intracellular lipids, favoring adipose tissue to accumulate mainly at the extracellular level, that is, between skeletal muscle fibers (intermuscular adipose tissue). In bone tissue, physical exercise favors mineralization and therefore reduces bone resorption. At the level of the immune system, physical exercise favors an increase in immune cells in the bloodstream, but also in cytokines and proteins of the acute inflammatory phase (e.g.,). In the cardiovascular system, physical exercise improves various cardiac parameters, and favors the release of diffusible factors that induce vasodilation. On the respiratory–pulmonary system, physical exercise increases alveolar ventilation and pulmonary blood flow. The impact of physical exercise on the gastrointestinal system is dependent on the intensity of this physical exercise. At low or moderate intensities, there are no changes (=) neither in motility nor in gastrointestinal absorption, while with intense physical activity there is a decrease in motility and an increase in gastrointestinal absorption. The endocrine system is also influenced by physical exercise. During the hyperactivity phase, an increase in the levels of various hormones is observed, which suggests that various hormonal axes are highly activated in this hyperactivity phase. On the contrary, in the phase of hypoactivity, the secretion of many hormones is interrupted. For details, see the main text. Source for figure illustrations: https://scidraw.io/ (accessed on 8 September 2021).

**Figure 4:**
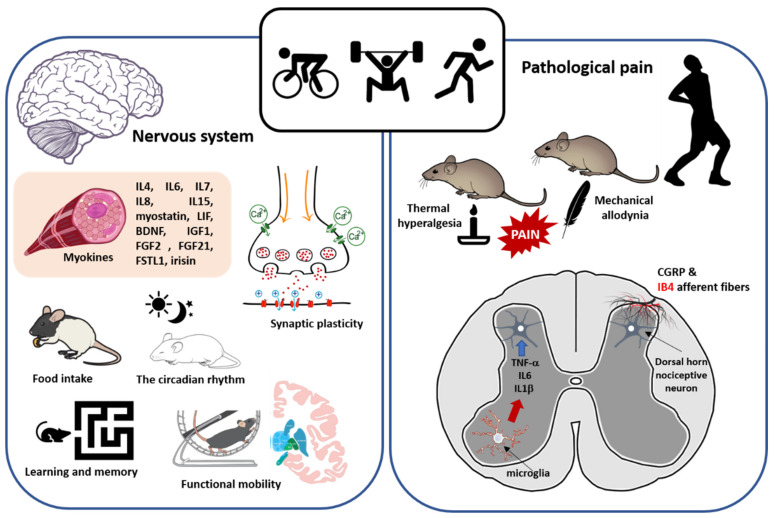
Effects of physical exercise on the nervous system and pathological pain. Various functions of the nervous system are also affected by physical exercise. Thus, physical exercise enhances synaptic plasticity, regulates food intake, modulates circadian rhythm, enhances learning and memory processes, and modifies neural circuits (e.g., basal ganglia) that improve the functional mobility of subjects. It should be noted that with physical exercise, the continued contraction of skeletal muscle fibers produces a significant release of myokines by this tissue. These myokines, together with other diffusible factors secreted by glial and vascular elements of the nervous system, are responsible for the changes described with physical exercise. On the other hand, physical exercise also has an impact on pathological pain. In animal models, physical exercise has been shown to reduce thermal hyperalgesia and mechanical allodynia. This analgesic effect of physical exercise is mediated by less activation of glial cells (e.g., microglia), which secrete fewer inflammatory cytokines, and with this there is less sensitization and excitation of the nociceptive neurons of the dorsal horn of the spinal cord, but also from nociceptive neurons from other supraspinal pain processing centers. At the spinal level, physical exercise also produces less branching of nociceptive peptidergic (CGRP-positive) and non-peptidergic (IB4-positive) afferent fibers in the dorsal horn of the spinal cord; therefore, there is less input of pain. For details, see the main text. Source for figure illustrations: https://scidraw.io/ (accessed on 8 September 2021).

Physical exercise also has an influence on other tissues other than adipose in both obese and non-obese subjects, including skeletal muscle tissue, bone tissue, and the immune system, as well as other systems such as cardiovascular, respiratory, gastrointestinal, endocrine, and nervous system. In the following sections, the physiological effects of physical exercises on body systems are discussed. In addition, Figure 3 and Figure 4 schematically illustrate such effects.

### 4.1. Skeletal Muscle

After physical exercise, an increase in the respiratory capacity of the mitochondria of skeletal muscle fibers is observed, as well as the mitochondrial content of citrate synthase [564,565]. Physical exercise (e.g., endurance training) also produces a reorganization of muscle adipose tissue since intramuscular adipose tissue is reduced and intermuscular tissue increased [566], regulates the processes of mitochondrial fusion and fission, increases type IIa fiber type with increased mitochondria and type IIa myosin heavy chain, and improves capacity of skeletal muscle blood flow by increasing VEGF [567]. Moreover, exercise (e.g., treadmill walking, jogging, cycling) increases lipoprotein lipase (LPL) activity in skeletal muscles of sedentary adults, an enzyme implicated in the metabolism of lipids, transforming triglycerides in free fatty acids [568].

Obesity is associated with numerous changes in skeletal muscle including greater muscle mass and muscle fiber cross sectional area, yet fasted muscle protein synthesis is lower. IGF-1 is a growth factor that regulates both anabolic and catabolic pathways in skeletal muscles, including pathways that regulates muscle mass maintenance, muscle hypertrophy, and muscle protein regulation [569]. However, it was reported that subjects with obesity have lower skeletal muscle IGF-1 mRNA and protein, at rest and following resistance exercise training [570]. After cycling activity during 45 min, it was reported that citrate synthase activity and maximal ATP production rate increase in non-obese, but not in obese, subject muscle samples [571]. However, the fatty acid oxidation in muscles increases after exercise training in extremely obese subjects with impaired fatty acid oxidation, at similar levels observed in lean subjects [572]. It was also reported that endurance exercise training upregulates fat oxidative capacity and lipolytic protein expression in skeletal muscle of obese subjects [573].

It should be noted that, in the obese subjects, several endocrinological disturbances have been identified during acute endurance and resistance exercise training including a blunted blood growth hormone, atrial natriuretic peptide and epinephrine release, and greater cortisol and insulin release. All of these endocrinological changes have an impact on the physiology of the skeletal muscle. Specifically, it has been observed that during acute endurance and resistance exercise, blood epinephrine release in obese subjects is smaller in comparison to healthy subjects. This hormone is implicated in lipolytic muscle response. Blood growth hormone secretion is significantly reduced in obese during acute endurance/resistance training, as opposed to healthy subjects. Growth hormone is implicated in the stimulation of skeletal muscle protein synthesis, by facilitating amino acid transport and availability via the release IGF-1 and other local factors. In contrast to healthy subjects, during acute endurance exercise, a greater blood cortisol levels seems to be present in the obese subjects. In skeletal muscle, cortisol causes muscle mass loss by a combination of a suppression of protein synthesis and increased protein degradation. Blood insulin levels also decrease in obese subjects after acute endurance training. This hormone stimulates skeletal muscle protein synthesis. Blood atrial natriuretic peptide levels do not increase with sufficient magnitude in subjects with obesity after acute endurance exercise, contributing to a suppression of lipolysis during exercise [574].

On the other hand, hyperinsulinemia in overweight/obese subjects is associated with increased extremity muscle mass, which is partially reversible with reduction in fasting insulin concentration, consistent with the stimulatory effects of insulin on skeletal muscle after treadmill exercise [575]. It is well known that insulin stimulates glycogen synthase (GS) through dephosphorylation of serine residues, and this effect is impaired in skeletal muscle from insulin-resistant obese subjects. It has been observed that 40 min of moderate-intensity cycle exercise increases GS fractional activity, decreases K(m) for UDP-glucose, and decreases GS phosphorylation. These findings suggest that the molecular regulatory process by which exercise promotes glycogen synthesis in muscle is preserved in insulin-resistant obese subjects [576].

### 4.2. Bone Tissue

The effect of physical exercise on the bone has been well studied in women. A recent meta-analysis study indicates that the exercise intervention resulted in significant, but rather small positive effect on bone mineral density (BMD) [577]. On the other hand, exercise downregulates sclerostin expression by the osteocyte favoring osteoblastogenesis. These changes are enhanced by dynamic cyclical load with rest periods and may be promoted by low-amplitude high-frequency stimuli [578]. The expression of osteoprotegerin increases after endurance exercise, but this increase is independent of the exercise intensity; however, the accumulation of collagen in the bone depends on the intensity of the exercise. Nevertheless, increased bone-alkaline phosphatase concentrations suggest a beneficial effect of this type of exercise on bone mineralization [579]. Actually, in humans, it was reported that resting femoral bone blood flow increases by physical exercise, but appears to level off with increasing exercise intensities [580]. In addition, it has also been observed that resistance exercise training increased markers of bone formation, while it transiently suppressed a marker of bone resorption, and adaptive changes of bone metabolism to resistance exercise training occurred during the early period of the training, before changes in bone density were observable through densitometry [581].

In rats, strenuous exercise causes a reduction of longitudinal and circumferential growth of the tibia, but the average number of osteons and osteocytes per unit area of the tibial middiaphysis was significantly greater in the exercised group. The number of osteons and osteocytes per unit area in the second metatarsus, however, was significantly less in the exercised group [582]. Other beneficial effects on bone tissue were observed in zebrafish subjected to increased physical exercise for four weeks in a swim tunnel experiment since showed: (i) a higher bone volume in the caudal spine; (ii) increased bone formation in posterior caudal vertebrae; (iii) the bone formation rate (BFR, amount of new bone formed in unit time per unit of bone surface) was 46% higher in the exercise group compared to the control; (iv) osteoblastic bone formation was significantly higher in the exercise group; and (v) higher mineralized bone in the exercise group. These finding suggest that controlled swimming exercise induces bone formation and mineralization in adult zebrafish [583].

### 4.3. Immune System

Several studies revealed that a single acute bout of prolonged, strenuous exercise has a temporary depressive effect on immune function. Actually, an acute bout of physical activity is accompanied by several responses of immune system, including an increase of circulating lymphocytes and neutrophils, pro-inflammatory cytokines (e.g., TNF-alpha, MCP-1, IL-1beta) and acute phase proteins (e.g., C-reactive protein) and under these circumstances, an increase of hormones (e.g., adrenaline, cortisol, growth hormone, prolactin) with immunomodulatory effects also were shown in plasma. Acute exercise temporarily increases the number of circulating natural killer (NK) cells, but following exercise, normal resting values are usually restored within 24 h. Antigen-presenting cell function is also affected by exercise. Exercise-induced reductions in macrophage major histocompatibility complex class II expression and antigen-presenting capacity. T-memory and T-naïve cells increases temporarily during exercise whereas the production of immunoglobulins by B-lymphocytes is inhibited following prolonged, strenuous exercise [583,584,585,586]. On the other hand, chronic exercise causes a slight increase of NK cell activity. Neutrophil function is suppressed or not significantly influenced by chronic exercise, whereas lymphocyte proliferative responses has been described as decreased, elevated or unchanged after chronic exercise [586].

In obese subjects, aerobic exercise and resistance exercise causes an increase of NK cell activity. Plasma levels of IgA remain stable, whereas IgG levels tend to decrease after training [587]. Several studies showed that leukocyte and lymphocyte subsets are elevated in obese individuals [588], but other studies indicate a reduction of lymphocytes in obese patients [589]. Obese animals also showed T-lymphopenia [590], and NK cell activity is suppressed [591].

### 4.4. Cardiovascular System

Regarding to cardiovascular system, dynamic physical exercise improves several hemodynamic and cardiac parameters (e.g., heart rate, stroke volume, cardiac output, total vascular conductance) in obese subjects [592]. At a vascular level, exercise increases the activity of endothelial eNOS an enzyme that synthetizes nitric oxide, a chemical mediator that induces vasodilatation [593] and consequently, exercise training consistently induces improvements in both conduit and resistance artery NO-mediated function. In addition, exercise also causes an increase in arterial cross-sectional area, also referred to as “arterial remodeling” [594]. Several cardiovascular adaptations were reported during exercise such as increase of cardiac output, heart rate, blood pressure, and reduction of pulmonary and systemic vascular resistance to blood flow [595,596]. An increase of cardiac output by exercise may be attributable to morphological changes of the heart, such as left ventricular dilatation and hypertrophy, leading to several physiological changes, including increase early diastolic filling, preload and myocardial relaxation, and also increase contractile strength [596,597]. The reduction of vascular resistance was also seen in several organs, such as skeletal muscle during exercise [598,599]. The hypertrophy of left ventricular is primarily the results of an increase in the size of cardiac myocytes [600]. The effect of exercise on cardiomyocyte contractile function may be related to alterations in the rise and decay rates of intracellular Ca^2+^ transients, possibly due to enhanced coupling efficiency between L-type Ca^2+^ channel-mediated Ca^2+^ entry and activation of subsarcolemmal ryanodine receptors (RyR; e.g., calcium-induced calcium release), and increased expression and activity of the sarcoendoplasmic reticulum Ca^2+^ ATPase (SERCA2a) and sodium-calcium exchanger (NCX) [601,602,603]. Reduction of vascular resistance to blood flow may be related to the release of vasodilatory signals (e.g., adenosine, lactate, potassium ions, CO_2_) [604,605,606].

### 4.5. Respiratory System

During exercise, pulmonary ventilation must rise in proportion to increase in metabolic rate so that arterial blood gas homeostasis is maintained, which it involves substantial increases in the work of breathing and thus the energy cost of breathing. However, the work of breathing is minimized, due to decreased resistance of the intra- and extra-thoracic airways during exercise. With exercise, pulmonary blood-flow and pulmonary arterial pressure rise whereas pulmonary vascular resistance falls. The dominant mechanism regulating pulmonary hemodynamic responses during exercise is left ventricular filling pressure, which climbs during exercise, and both recruits and distends the pulmonary capillaries and veins, thus contributing to the fall in pulmonary vascular resistance. Furthermore, vascular distention and recruitment expand the capillary surface area and volume, thus minimizing any fall in red blood cell transit time within the pulmonary capillaries in the face of an increasing cardiac output. Interstitial and alveolar edema is minimized because both capillary absorptive pressure and lymph flow increase during exercise. During exercise, the efficiency of gas exchange worsens in an intensity-dependent manner, but a disproportionate rise in alveolar ventilation relative to metabolic rate during high-intensity exercise prevents arterial hypoxemia [607].

Obese patients show a decline in exercise capacity and diverse degrees of dyspnea in association with mechanical abnormalities, increased ventilatory requirements secondary to the increased metabolic load, and a greater work of breathing. Consequently, obese patients may be particularly predisposed to the development of respiratory muscle fatigue during exercise. In this context, it was reported that in obese subjects the tidal volume, the breathing frequency, the power output, and the peak oxygen uptake were significantly higher after training. At comparable workload, training induces lower minute ventilation, mouth occlusion pressure, ratio of occlusion pressure to maximal inspiratory pressure and rate of perceived breathlessness. These findings suggest that aerobic exercise at ventilatory threshold may induce significant improvement in respiratory muscle strength, maximal exercise capacity, and inspiratory muscle performance and decreased dyspnea perception in obese subjects [608]. Despite these results, it should be noted that mechanical ventilatory constraints increase progressively with degrees of obesity, contributing to exercise limitation in obese subjects [609,610].

On the other hand, one of the mechanisms related to respiratory mechanics alteration caused by obesity is the accumulation of fat in chest, diaphragm, and abdomen [611] since this accumulation may compress the chest wall, diaphragm, and lungs, reducing lung volumes and flow [612]. Consequently, this accumulation of fat may affect several spirometry and respiratory parameters. It was reported that, in obese subjects, the basal values of blood pressure and heart rate were higher whereas oxygen saturation was lower in comparison to eutrophic group. During exercises, oxygen saturation also decreased during exercise, whereas blood pressure and heart rate were higher. With respect to spirometry variables, FVC and FEV1 were lower in obese subjects before and after the exercise. These findings suggest that obesity affects pulmonary function [613] and reduction of functional residual capacity and impairment of diffusion capacity were reported in obese subjects [611]. Finally, in non-asthmatic subjects, the airway responsiveness was significantly correlated with BMI, and this increase was significantly related to lung volume restriction [614].

### 4.6. Gastrointestinal System

Gastroesophageal reflux occurs more frequently with exercise than at rest and may produce symptoms of chest pain suggestive of ischemic disease. Acid exposure may be reduced by pretreatment with histamine H2-receptor antagonists. Gastric emptying during exercise is subject to a number of factors including calorie count, meal osmolality, and meal, temperature, and exercise conditions. It seems that moderate exercise would have little effects on gastrointestinal tract motility, but when exercise becomes more severe, there may be some inhibiting effects, especially at the level of gastric emptying. Gastric acid secretion probably changes little with exercise although it has been postulated that ulcer patients may increase secretion with exercise. Some exercise-associated digestive symptoms, such as diarrhea and abdominal pain, have been attributed to changes in intestine function. Small bowel transit is delayed by exercise when measured by breath hydrogen oral cecal transit times and motility may be reduced as well. Intestinal absorption during exercise has not been well evaluated, but it likely changes little in ordinary circumstances. Passive absorption of water, electrolytes, and xylose are not affected by submaximal effort, but increased intestinal permeability was reported after a marathon. After higher intensities of exercise, gastrointestinal changes include a reduction in mesenteric blood flow, decreases in esophageal peristaltic activity, decreases in lower esophageal sphincter tone, and increased transient lower sphincter time and a reduction of gastric emptying. Due to reduced gastrointestinal tract perfusion, absorption might be affected and gut barrier function might be compromised [615,616].

As for obese subjects—several studies suggest that physical exercise causes changes in the gastrointestinal system including liver. It was reported that exercise decreased late-phase postprandial gallbladder volume and increased late-phase postprandial gallbladder motility in obese subjects [617]. On the other hand, endurance exercise training reduces plasma cholesterol despite increasing cholesterol absorption in subjects with obesity and metabolic syndrome risk factors [618]. Endurance training did not decrease hepatic fat in obese subjects, but training lowered plasma liver enzymes (e.g., GGT), but not ALT and bilirubin [619]. However, other studies indicate that in obese subjects, blood ALT and AST tend to decrease after endurance-strength training, whereas both enzymes increase after endurance training. On the other hand, a significant reduction in serum of GGT was seen in both trainings and neither endurance nor endurance-strength exercise led to changes in serum ALP activity and total or direct bilirubin level. These findings suggest that the mode of exercise does matter: endurance-strength exercise led to a greater improvement, compared to endurance exercise, in the liver functioning in subjects with abdominal obesity [620]. Finally, no studies have assessed the impact of physical exercise on motility, digestion, and absorption processes of the gastrointestinal system in obese subjects.

### 4.7. Endocrine System

Hormonal changes related to exercise may be explained by several physiological reasons: (i) to induce cardiovascular adjustments; (ii) to activate energy production pathways and mobilize energy substrates; (iii) to facilitate maintenance of adequate hydration; and (iv) to some extent as part of stress reactivity. During exercise, the endocrine system hormonal responses seem to occur in two phases: an initial hyperactivity phase, followed by a latter hypoactivity phase. In the hyperactivity phase, elevations in the circulating levels of hormones, such as adrenocorticotrophic hormone, cortisol, prolactin, and catecholamines were reported at rest and/or in response to an acute exercise session. In the hypoactivity phase certain hormones including adrenocorticotrophic hormone, catecholamines, cortisol, growth hormone, follicle-stimulating hormone, luteinizing hormone, testosterone, and thyroid hormones are found suppressed. In this hypoactivity phase, several inflammatory mediators (e.g., IL6, TNF-alpha, and UL-1beta) act upon multiple levels of the hypothalamic-pituitary-adrenal axis, most notably the hypothalamic paraventricular nucleus where CRF production occurs. CRF influences this endocrine axis, but also affect mood, sexual, and immune functions [621]. Growing evidences suggest that exercise and training affect the hypothalamic-pituitary-adrenal (HPA) axis. In males, testosterone increases with acute bouts of exercise, but lower testosterone levels were seen in endurance athletes. In females, an increase of total testosterone levels was seen after an acute bout of resistance training and multiple studies have found an acute increase in estradiol immediately after exercise [622]. On the other hand, exercise stimulating HPA axis also induces elevation of cortisol, corticosterone, and catecholamines in plasma. Glucocorticoids play an important role in the mobilization of energy reserves during physical activity by stimulating gluconeogenesis, promoting lipolysis and increasing protein catabolism, whereas catecholamines secreted from the adrenal medulla stimulates the release of IL6 by immune cells. IL-6 is known to stimulate two other hormones: growth hormone (GH) and prolactin (PRL) [623]. Finally, several studies revealed that in diabetic subjects, exercise decrease pancreatic fat content, and increase the release of insulin by endocrine pancreas [624,625,626,627], a key hormone in the regulation of sugars, lipids, and amino acids after food intake.

In older obese subjects, it was reported that exercise training increases beta-cell function. The increase in pancreatic function is clinically important, since it was directly related to improved glucose tolerance [628].

### 4.8. Nervous System

Physical exercise induces different changes in several neuronal nuclei of the central nervous system. In locus coeruleus (LC) involved in the regulation of attention, arousal, vigilance responses to stress, and spinal cord modulation of pain, exercise causes level variation of galanin, a neurotransmitter attenuates neuronal hyper-excitability. Exercise may therefore be involved in the LC noradrenergic neurons adaptation to stress. Moreover, exercise increases serotonin in the suprachiasmatic nucleus (SCN), the hypothalamic nucleus involved in the control of the circadian rhythm or circadian clock. The circadian clock is a timing mechanism that endogenously coordinates biochemical, physiological, and behavioral processes with the 24 h cycle of light and dark. Moreover, physical exercise also influences hypothalamic neurons involved in regulating food intake. That is, the acute exercise reduces the food intake by activation of the corticotropin-releasing factor (CRF/CRH) pathway in the dorsomedial hypothalamus, and increases leptin levels, whereas chronic exercise inhibits food intake by a pathway leptin-independent. In the basal ganglia, clinical studies demonstrated that exercise might be involved in improving functional mobility of patients with Parkinson’s disease since it increases brain derived neurotrophic factor (BDNF), a growth factor with an important role for the survival of dopaminergic neurons in the striatum [629]. In addition, it is well known that regular exercise has a beneficial impact on depression, quality of sleep, and cognitive function. Chronic physical activity increases the expression of brain growth factors, such as BDNF and VEGF, factors that are involved in enhancing the learning process by regulating synaptic plasticity, or in improving cerebral vascularization [630,631]. Training skeletal muscle contractions stimulates the production and release of myokines in the blood, including IL4, IL6, IL7, IL8, IL15, myostatin, leukemia inhibitory factor (LIF), brain-derived neurotrophic factor (BDNF), insulin-like growth factor (IGF1), fibroblast growth factor 2 (FGF2), fibroblast growth factor 21 (FGF21), follistatin-related protein 1 (FSTL1), irisin, erythropoietin (EPO), β-aminoisobutyric acid (BAIBA), CTRP15 (myonectin), and decorin, which can influence physiology of the nervous system [632]. IL4 modulates the expression of pro-inflammatory cytokines, nitric oxide, and growth factors in astrocytes and microglia cells of the central nervous system, decreasing the secretion of inflammatory mediators and promoting the release of growth factors. Likewise, IL4 participates in the survival of hippocampal and striatum neurons, and reduces pain caused by subsequent intraplantar injection of carrageenan, bradykinin, or TNF-α [633]. IL8 acts in the brain by reducing food intake [634]. IL15 provides neuroprotection to various types of neurons, but at the same time, causes reactivation of microglia cells and astrocytes. In the hypothalamus it regulates temperature, energy metabolism, and circadian rhythms, whereas in hippocampus promotes consolidation of learning and memory [635]. As for IGF-1, it promotes plasticity at synapse levels, such as increasing the length and number of multiple spin bouton complexes as well as the length of the post-synaptic density in hippocampal neurons. Moreover, it increases expression of synapsin-1, a protein important for maintaining the stability of synapses. IGF-1 is also implicated in the modulation of glutamatergic receptor subunits, alterations in calcium channel conductance as well as potentiation of glutamatergic transmission, in the face of a reduction in GABAergic transmission [636]. On the other hand, different brain regions are sensitive to the action of FGF21 including the suprachiasmatic nucleus, area postrema, nucleus of the solitary tract and paraventricular nucleus of the hypothalamus, and smaller amounts in the ventral tegmental area and nucleus accumbens. FGF21 reduces sugar intake in both mice and humans, and regulates alcohol preference [637]. Regarding irisin, it improves learning and memory by increasing BDNF expression in hippocampus [638]. Finally, EPO plays different roles in the nervous system including survival and neuroprotection, modulation of the inflammatory and immune response, hemodynamic, and vasoactive effects, promotes angiogenesis for stimulating mitosis and motility of endothelial cells [639,640].

Finally, it has been shown that regular training increases autonomic nervous system activity in obese subjects [641,642]. Exercise intervention is associated with attenuation in the response to visual food cues in brain regions known to be important in food intake regulation, such as the insula [643]. Aerobic exercise causes a significant increase in plasma BDNF levels in obese subjects [644,645]. A similar pattern was reported after acute high-intensity interval exercise in obese subjects [646].

### 4.9. Pathological Pain: Relationship with Sedentary Behavior and Modulation by Physical Exercise

Sedentary behavior (SB), defined as activities that do not increase energy expenditure substantially above the resting level (e.g., 1.0–1.5 metabolic equivalents), is characterized by waking time spent sitting, reclining, or lying down [647,648]. Globally, adults spend a considerable percentage of their daily time in SB, such as workplace sitting, television watching, or computer and game-console use [649]. Time spent watching TV, in particular, is positively correlated with increasing body mass index (BMI) [650,651,652]. There is evidence that increased obesity risk is attributed to time spent in sedentary transportation (e.g., car, train, bus) [653,654]. In contrast, it was also reported that greater leisure time moderate-to-vigorous leisure time physical activity (MVPA) (e.g., sports and exercise), active transportation (e.g., walking and bicycling), and reading were independently related to lower BMI, whereas greater TV/movie watching, games, computer use, and sedentary transportation were independently related to higher BMI. However, there were statistical interactions between leisure time MVPA and TV/movie watching, leisure time MVPA and playing games, active transportation and sedentary transportation, and active transportation and reading in relation to BMI. These findings suggest that different combinations or patterns of physical activity and sedentary behaviors may need to be considered in terms of their implications for BMI risk [655].

Cross-sectional and longitudinal research has found that, among healthy adults or adults with chronic pain, individuals with a higher level of SB report a higher level of pain than those who are less sedentary [647,656,657]. It is well known that chronic pain is a highly prevalent and extremely debilitating condition that places a burden on daily living and decreases life expectancy. Lifestyle factors such as physical inactivity, stress, poor sleep, unhealthy diet, and smoking are associated with chronic pain severity and sustainment. This applies to all age categories, that is, chronic pain across the lifespan [658]. Knee osteoarthritis (OA) are the primary cause of chronic pain in older adults. Using a sample of older knee osteoarthritis (OA) patients, it was reported that patients spent less time in moderate-to-vigorous physical activity (MVPA), but more time in sedentary behavior (SB) on days when they endorsed greater levels of catastrophic thinking about pain in the morning. The predictive effects of a patient’s pain catastrophizing also extended to sedentary behavior the next day. In addition, patients reported greater pain catastrophizing in the morning if they spent more time than usual in sedentary behavior the previous day, suggesting that pain catastrophizing could be worsened by avoiding physical activities. These results provide strong evidence to support the hypothesized role of pain catastrophizing in affecting pain patients’ physical activity in everyday life, being able to exist a vicious circle between catastrophic pain and sedentary behavior, in other words, one enhances the other and vice versa [659]. Longer sedentary behavior was correlated with chronic knee pain, and subjects with high levels of physical activity were less likely to suffer from chronic knee pain [660]. On the other hand, in severely obese adult subjects (with BMI ≥ 35 kg/m^2^), a significant association between pain and MVPA (*p* = 0.0017) was reported, indicating that individuals with shorter MVPA have more pain. However, no association was seen between pain and light physical activity (LPA) or SB. These findings indicate that, in severely obese individuals, shorter MVPA time is associated with a higher prevalence of pain [661]. Positive associations with daily SB were found for pain intensity (r = 0.31, *p* < 0.01) and the number of painful joints (r = 0.24, *p* < 0.05), but not non-articular pain in individuals with rheumatoid arthritis [662]. High levels of sedentary time and lower levels of light physical activity are associated with a worse symptomatology in fibromyalgia [663]. All of these studies suggest the existence of a relationship between sedentary behavior and pain, in pathologies that, according to the International Association for the Study of Pain (IASP), trigger pathological pain.

There is scientific evidence that suggests that physical exercise improves pain in subjects with sedentary behavior and pathological pain diseases. Two-month intensive aquatic therapy program of high frequency (five times/week) decreases levels of back pain and disability, increases quality of life, and improves body composition and health-related fitness in sedentary adults with chronic low back pain [664].

In addition, several studies suggest beneficial effects of physical exercise on pain responses in subjects suffering from pathologies that trigger pathological pain. It was reported that compared to the control group, three exercise modalities (e.g., Tai Chi, Baduanjin, and stationary cycling) significantly increased knee injury and osteoarthritis outcome score pain subscores and regulated serum PD-1 and INF-γ levels in patients with knee osteoarthritis [665]. Isometric exercise in patients with type 2 diabetes mellitus with and without painful diabetic neuropathy promotes exercise-induced hypoalgesia (EIH) [666]. Aerobic exercise [667] muscle strengthening, pool exercises [668], and spa therapy [669] have been found to improve the pathology in subjects with fibromyalgia, including pain. Fibromyalgia patients can attain symptom relief, particularly decreased pain and fatigue as well as improved sleep and mood, with low-to-moderate intensity exercise of any type [670].

Experimental and clinical evidence suggest that physical exercise influences pathological pain, both neuropathic and nociplastic. Studies evaluating the effect of exercise after spinal cord injury indicate that the intervention significantly improves mechanical allodynia and thermal hyperalgesia in injured subjects [671]. In rats subjected to spinal cord contusion, exercise training in treadmill causes an upregulation of BDNF expression, and this is associated with greater thermal hyperalgesia and mechanical allodynia [672]. In addition, after spinal cord contusion, all rats showed mechanical and thermal hypersensitivity, and exercise causes a reduction of animals with mechanical allodynia. Exercise also reduces the sprouting of IB4-positive afferent fibers in the dorsal horn of injured rats [673] and it alleviates hyperalgesia in rats subjected to spinal cord compression [674]. Furthermore, after spinal cord contusion in mice, exercise alleviates hyperalgesia and reduces CGRP-immunoreactivity in dorsal horn [675]. In the same line, chronic exercise training also alleviates mechanical and thermal hyperalgesia in mice subjected to spinal cord compression [676].

On the other hand, after chronic constriction injury (CCI) of the sciatic nerve, exercise alleviates thermal and mechanical hyperalgesia by reducing the expression of pro-inflammatory cytokines in rats (e.g., TNF-alpha, IL6) [677]. Transcranial direct current stimulation combined with exercise also alleviates both mechanical and thermal hyperalgesia and modulates the expression of pro-inflammatory cytokines in several central nervous system areas in rats [678]. The combination of ultrasound and exercise also alleviates hyperalgesia and reduces the expression of pro-inflammatory cytokines, and improve the expression of anti-inflammatory cytokines in rats [679]. It is worth mentioning that exercise reduces irisin expression in rats subjected to CCI [680].

After spared nerve injury (SNI), exercise alleviates both mechanical and thermal hyperalgesia [681], and this pattern was seen in mice subjected to partial ligation of sciatic nerve, where exercise also reduces gliosis [682]. In this experimental model of partial ligation of sciatic nerve, it was also reported that exercise alleviates hyperalgesia and causes a significant reduction of inhibitory GABAergic neurons in the dorsal horn [683].

As for nociplastic pain, it has been shown that resistance exercise improves muscle strength, health status and pain intensity in women with fibromyalgia [684] and spinal stabilization exercise (SSE) plus kinesio taping (KT) improve pain responses in patients with this health concern [685]. Moreover, exercise in warm water decreases pain and improve cognitive function in subjects with fibromyalgia [686]. Actually, it was reported that physical exercise tends to reduce pain in patients with fibromyalgia and this effect is associated with a decrease in IGF-1 levels in the cerebrospinal fluid of these patients [687]. Hence, low to moderate intensity global exercises performed for a long period of treatment should be performed in patients with nociplastic pain predominance. Additionally, focused and intense exercises for a short period of treatment can be prescribed for patients with nociceptive pain predominance [688].

It should be noted that in the context of pain, inflammatory cytokines (e.g., TNF-alpha, IL6, and IL1beta) as well as BDNF, and IGF-1 causes hyperalgesia for: (i) stimulating nociceptive neurons and causing hyperexcitability, and (ii) inducing central sensitization [689,690,691]. However, irisin has anti-inflammatory properties by attenuating the expression of pro-inflammatory cytokines [692] and their intrathecal administration alleviates hyperalgesia after CCI [693]. However, it is well known that irisin promotes the expression of BDNF [694], a growth factors that may cause pain behaviors.

Finally, few studies have focused on studying the effects that exercise exert on pain in obese subjects. It has been shown in mice that high-fat diet induces prediabetes, causing hyperalgesia and exercise attenuates this pain response in obese mice. Actually, obese prediabetic mice showed higher levels of GDNF and NGF in nerves and spinal cord [695]. In contrast, swimming does not alter nociception threshold in obese rats submitted to median nerve compression [696]. Regarding human studies, it has been suggested that increased physical exercise has no mitigating effects on low back pain in obese subjects [49], but further studies on the beneficial effects of physical exercise are needed to obtain robust conclusions on pain modulation on obese subjects.

## 5. Conclusions

A sedentary lifestyle leads to the development of overweight/obesity, which involve excessive fat body accumulation. This fat accumulation may trigger several pathophysiological comorbidities, which would result from adipose tissue and body systems crosstalking. Our review offered an overview of structural and functional changes in tissues, organs, and body systems due to the crosstalk between fat and body tissues, and how physical exercise may modulate fat accumulation-related comorbidities, including pathological pain development. To conclude, firstly, the literature review provided evidence that in overweight/obese subjects, the following is present: adipose tissue and other body tissue crosstalk mediated by diffusible factors with local or generalized effects, occurring mainly on skeletal muscle, cardiovascular system, liver, pancreas, and visceral, abdominal, and subcutaneous adipose tissues. Consistent with the literature, we showed that, besides these general body system pathological changes, neuropathic and nociplastic pain development might be associated with overweight/obesity. Concretely, it has been evidenced that in obese subjects, there is a powerful crosstalk between body organ adipose tissue and the nociceptive afferent nerve fibers present in these organs; diffusible factors secreted by the adipose tissue would lead to nociceptor sensitization and hyperexcitability, resulting in pain development. On the other hand, several beneficial effects of physical exercise has been evidenced, not only on adipose tissue, but also on organs and body systems, including skeletal muscle, bone tissue, immune system, cardiovascular system, respiratory-pulmonary system, gastrointestinal system, endocrine system, and the nervous system. That is, physical exercise not only favors a reduction in body mass with a direct impact on decreasing overweight/obese-induced comorbidities associated with adipose tissue factors released, but it also directly affects other organs and tissues. Finally, focusing on pathological fat accumulation-related pathological pain, the literature allowed describing experimental and clinical evidence, suggesting that physical exercise may modulate both neuropathic and nociplastic pain symptoms. However, it has been evidenced that further studies on the beneficial effects of physical exercise are needed to obtain robust conclusions about pain modulation, specifically in obese subjects.

## Figures and Tables

**Table 1:** Diffusible factors synthesized and released by adipose tissues in obese subjects ^1^.

Factor/Molecule	EAT	HAT	IMAT	PAT	PPAT	PVAT	VAT/SAT
Adiponectin						X	X
Adrenomedullin						X	X
Angiotensin-II						X	
Apelin						X	
bFGF						X	
CCL1							X
CCL11	X						
CCL18	X						
CCL21	X						
CCL3	X					X	
CCL4	X						
CCL5	X					X	
CCL8						X	
Chemerin						X	
CTRP-9						X	
CXCL1					X		
CXCL10							X
CXCL2					X		X
CXCL3					X		
CXCL5/LIX					X		
CXCL9							X
FFA	X						
FGF-21		X					
Fractalkine							X
HGF						X	
H2O2						X	
H2S						X	
IGFB-3						X	
IL1-beta	X					X	X
IL10						X	X
IL12							X
IL13							X
IL15							X
IL17						X	
IL18							X
IL32							X
IL33							X
IL6	X					X	X
IL7							X
IL8							X
INF-γ							X
Irisin		X					
Leptin				X		X	X
Lipocalin-2						X	
MCP-1/CCL2	X		X			X	X
Methyl palmitate						X	
Micro-RNAs						X	X
MIP-1α/CCL3					X		
MIP1							
MIP3							
Nitric Oxide						X	
Omentin						X	
Osteopontin							
Osteoprotegerin							
PDGF				X		X	
RANTES						X	
Resistin	X			X		X	
ROS						X	
Serpin-E1						X	
TGF-β				X		X	
Thrombospondin-1						X	
TNF-alpha	X			X		X	
TWEAK							
Vaspin						X	
VEGF					X	X	
Visfatin						X	

^1^ EAT (Epicardial adipose tissue); HAT (Hepatic adipose tissue); IMAT (Intermuscular adipose tissue); PAT (Pericardial adipose tissue); PPAT (peri-pancreatic adipose tissue); PVAT (perivascular adipose tissue); VAT/SAT (visceral and subcutaneous adipose tissue). X: indicate diffusible factors synthesized/released by the adipose tissue of the corresponding column.
